# Dynamic actin cycling through mitochondrial subpopulations locally regulates the fission–fusion balance within mitochondrial networks

**DOI:** 10.1038/ncomms12886

**Published:** 2016-09-30

**Authors:** Andrew S. Moore, Yvette C. Wong, Cory L. Simpson, Erika L. F. Holzbaur

**Affiliations:** 1 Department of Physiology, Perelman School of Medicine, University of Pennsylvania, 638A Clinical Research Building, 415 Curie Boulevard, Philadelphia, Pennsylvania 19104, USA; 2 Department of Dermatology, Hospital of the University of Pennsylvania, Philadelphia, Pennsylvania 19104, USA

## Abstract

Mitochondria form interconnected networks that dynamically remodel in response to cellular needs. Using live-cell imaging, we investigate the role of the actin cytoskeleton in regulating mitochondrial fission and fusion. We identify cycling of actin filaments onto and off of subsets of cellular mitochondria. The association of actin filaments with mitochondrial subpopulations is transient; actin quickly disassembles, then reassembles around a distinct subpopulation, efficiently cycling through all cellular mitochondria within 14 min. The focal assembly of actin induces local, Drp1-dependent fragmentation of the mitochondrial network. On actin disassembly, fragmented mitochondria undergo rapid fusion, leading to regional recovery of the tubular mitochondrial network. Cycling requires dynamic actin polymerization and is blocked by inhibitors of both Arp2/3 and formins. We propose that cyclic assembly of actin onto mitochondria modulates the fission/fusion balance, promotes network remodelling and content mixing, and thus may serve as an essential mechanism regulating mitochondrial network homeostasis.

## Abstract (web summary)

Mitochondria are dynamic organelles that can undergo fission and fusion. Here the authors identify a novel pathway in which actin dynamically assembles in an Arp2/3- and formin-dependent manner around a subset of cellular mitochondria, promoting localized Drp1-dependent fission and impeding fusion.

## Graphical abstract

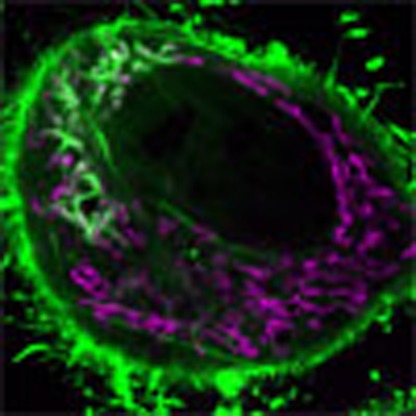

Mitochondria are dynamic organelles that undergo fission and fusion to segregate their content of DNA, facilitate transfer of mitochondrial proteins and enable mitophagic clearance of damaged organelles^1^,^2^,^3^. The steady-state balance between mitochondrial fission and fusion is a key determinant of overall mitochondrial network structure and, by extension, cellular bioenergetics^4^. The processes of mitochondrial fission and fusion are regulated by a collection of large GTPases. Mitofusin 1 and Mitofusin 2 control fusion of the mitochondrial outer membrane^5^, whereas Opa1 coordinates inner membrane fusion^6^. In contrast, Drp1, through association with specific mitochondria-localized receptors, drives fission of both inner and outer mitochondrial membranes^7^,^8^.

Before Drp1 recruitment, the endoplasmic reticulum (ER) marks prospective sites of mitochondrial fission^9^. ER tubules twist around mitochondria, inducing a pre-constriction event that decreases the mitochondrial cross-sectional diameter and allows for Drp1 assembly at the site of fission. The force necessary to drive this mitochondrial constriction is provided by actin polymerization by the ER-associated formin INF2 and the mitochondria-anchored formin-activating protein Spire1C^10^,^11^,^12^. Consistent with a key role for actin in the initial step of mitochondrial fission, actin depolymerization or depletion of crucial actin-polymerizing proteins results in increased mitochondrial length^10^,^13^. In addition, actin was shown to directly bind to and activate Drp1, and is robustly recruited to fragmenting mitochondria after treatment with chemical ionophores^13^,^14^.

Although the contribution of actin to mitochondrial fission has been examined at the level of the individual organelle, much less is known about how the actin cytoskeleton regulates the mitochondrial fission/fusion balance on a cell-wide level. To investigate this question, we used live-cell imaging to examine the dynamic interactions of actin with mitochondria. We made the unexpected observation that filamentous actin (F-actin) cyclically associates with distinct mitochondrial subpopulations in an Arp2/3- and formin-dependent manner, to regulate mitochondrial networks within the cell. Actin dynamically assembles onto the outer membranes of a subpopulation of healthy, elongated mitochondria to promote fission and to inhibit fusion. Following mitochondrial fission, actin disassembles from the fragmented mitochondria, which rapidly re-fuse and reintegrate into the mitochondrial network. Actin subsequently assembles around a distinct, often neighbouring mitochondrial subpopulation and the process repeats. Over 14 min, actin cycles through all mitochondrial subpopulations, locally enhancing mitochondrial dynamics, which facilitates network remodelling and content mixing. Our study thus highlights actin dynamics during mitochondrial fission/fusion and identifies actin cycling as a homeostatic regulator of cellular mitochondrial morphology.

## Results

### Actin is associated with mitochondrial subpopulations

**Figure 1:**
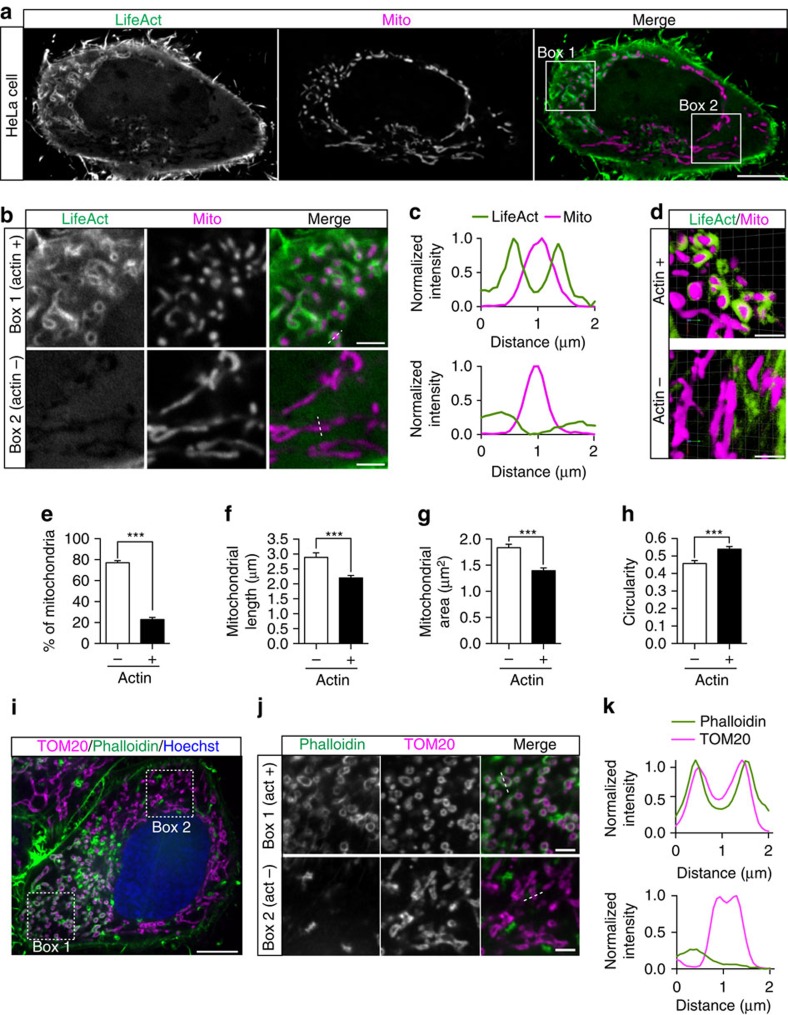
F-actin localizes to subpopulations of mitochondria. (**a**) Confocal microscopy image of F-actin (LifeAct-GFP) localization to a subset of Mito-DsRed2-labelled mitochondria in HeLa cells. (**b**) Enlarged images showing fragmented, actin-positive mitochondria (Box 1) and elongated, tubular mitochondria that have not recruited actin (Box 2). (**c**) Line scans of actin-positive mitochondria (top) and actin-negative mitochondria (bottom). (**d**) Three-dimensional renderings of *z*-stack images showing differential morphologies of actin-positive mitochondria (top) and actin-negative mitochondria (bottom) within the same cell. (**e**) Percentage of actin-positive and actin-negative mitochondria per cell. (**f**,**g**) Mitochondria that have recruited actin have decreased length and area. (**h**) Actin-positive mitochondria have a significantly higher circularity index than actin-negative mitochondria. (**i**) Confocal image of phalloidin recruitment to mitochondria labelled with anti-TOM20 antibody in a fixed HeLa cell. (**j**) Enlarged images from the boxed region in **i** displaying differential morphologies of phalloidin-positive and phalloidin-negative mitochondria. (**k**) Line scans of individual mitochondria from the regions shown in **j** showing co-localization of the TOM20 outer membrane marker with phalloidin (top) and TOM20 without actin co-localization (bottom). Values represent means±s.e.m. ***P<0.001. Scale bars, 10 μm (**a**,**i**); 2.5 μm (**b**,**d**,**j**). Sample size: (**e**) 19 cells, (**f**) 9 cells, (**g**) 10 cells, (**h**) 26 cells. *N*≥3 independent experiments in all cases.

To explore the dynamics of actin assembly on mitochondria, we imaged live HeLa cells expressing LifeAct-GFP, a marker that preferentially labels F-actin^15^, and Mito-DsRed2, a mitochondrial matrix marker. As expected, the majority of actin filaments localized to the cell periphery and did not associate with mitochondria (Fig. 1a). However, we were also able to observe F-actin localization to subpopulations of mitochondria (Fig. 1a, Box 1). Using confocal microscopy, we identified clear LifeAct-GFP rings surrounding individual mitochondria (Fig. 1b,c), demonstrating robust recruitment of actin to the outer membrane of these organelles. Three-dimensional renderings of confocal *z*-stacks revealed F-actin cages entirely surrounding subpopulations of cellular mitochondria (Fig. 1d).

At any point in time, we found that LifeAct was recruited to only a subset (on average 23%) of the total mitochondrial pool (Fig. 1e). To query the functional consequence of this cytoskeletal association, we compared the size and morphology of actin-positive and actin-negative mitochondria within cells. We found that mitochondria with associated actin were significantly shorter, smaller and less tubular (more circular) than mitochondria without associated actin (Fig. 1f,g,h).

In parallel experiments in untransfected fixed cells, we observed localization of endogenous F-actin (visualized using phalloidin) to TOM20-stained mitochondria in 95.2% of Hela cells (*n*=105 cells from three independent experiments; Supplementary Fig. 1). Within each cell, we identified F-actin assembly on 22% of mitochondria (Fig. 1i,j,k and Supplementary Fig. 2a). Consistent with our live-cell data, we found that phalloidin-positive mitochondria were comparatively more fragmented than those not associated with actin (Supplementary Fig. 2b,c,d). In fixed cells, visualization of F-actin recruitment to mitochondria is most apparent in single confocal slices, but is also clearly visible in maximum intensity projections of confocal stacks (Supplementary Fig. 2e).

### Actin cycles through mitochondrial subpopulations

**Figure 2:**
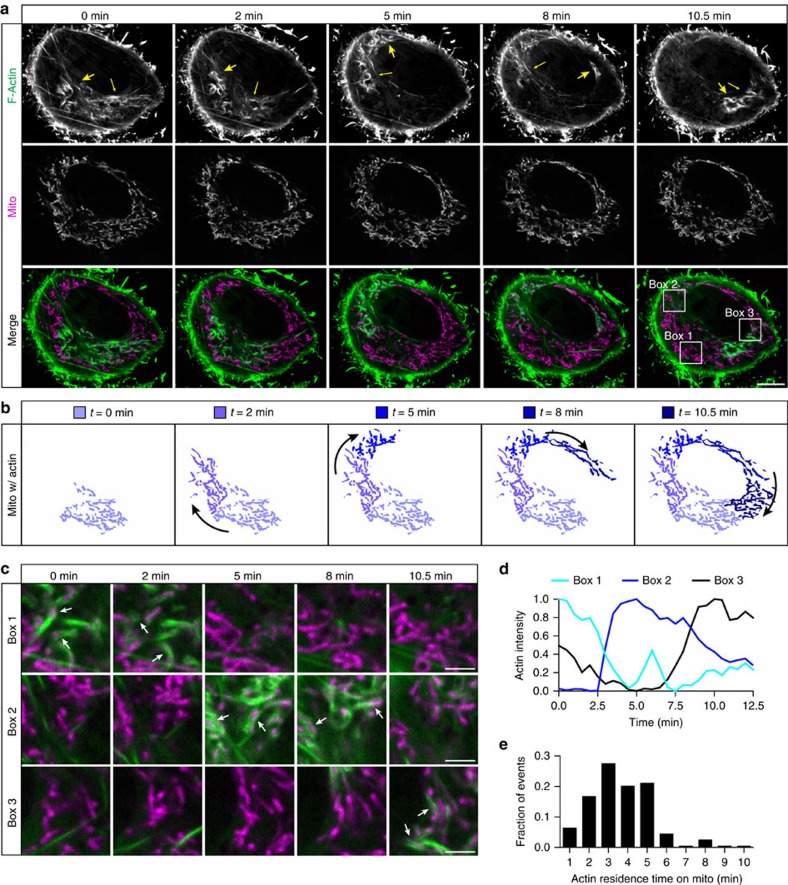
Actin cycles through mitochondrial subpopulations within the cell. (**a**) Confocal time series showing F-actin (LifeAct-GFP) cycling through different mitochondrial subpopulations within the cell in a clockwise direction over 10.5 min. Large arrow indicates leading edge of actin-positive mitochondria; small arrow indicates trailing edge of actin-positive mitochondria. (**b**) Model of actin cycling through mitochondrial subpopulations over time corresponding to (**a**). (**c**) Enlarged images of boxes 1–3, showing temporally ordered actin polymerization/depolymerization on individual mitochondria (white arrows). (**d**) Normalized actin intensities within boxes 1–3 over time. (**e**) Histogram of F-actin residence time on individual mitochondria during cycling. Values represent means±s.e.m. Scale bars, 10 μm (**a**); 2.5 μm (**c**). Sample size: (**e**) *n*=204 mitochondria from 22 cells; *n*≥3 independent experiments.

Over time, we found that actin filaments did not remain stably associated with the same subset of mitochondria. Instead, we observed actin cycling through different mitochondrial subpopulations. Imaging cells expressing LifeAct-GFP and Mito-DsRed2 over time shows the cycling of polymerized actin from one mitochondrial subpopulation to another (Fig. 2a–c, Supplementary Fig. 3 and Supplementary Movies 1–4). The assembly and disassembly of actin filaments around specific mitochondrial subpopulations is most clearly seen in enlarged images of specific regions of the cell (Fig. 2c) and the corresponding quantification of the overall fluorescence intensity of LifeAct-GFP within these boxed regions (Fig. 2d). Actin remained associated with each subpopulation of mitochondria for ∼3–5 min before depolymerizing (Fig. 2e). On depolymerization, actin subsequently repolymerized around a distinct, usually adjacent, subpopulation of mitochondria (the clockwise movement of the arrows in the time series shown in Fig. 2a and Supplementary Movie 1 is noteworthy), resulting in the robust cycling of actin through the entire mitochondrial network (see model in Fig. 2b). We observed this process of actin cycling in 86.8% of all cells (*n*=114 cells from 7 independent experiments; Supplementary Movie 5).

To confirm our initial observations of actin dynamics made using LifeAct-GFP, we performed similar imaging experiments using either GFP-actin (Supplementary Fig. 4 and Supplementary Movie 6) or F-tractin-GFP^16^ (Supplementary Movie 7), to visualize actin filaments. With all three probes, we noted very similar dynamics: actin transiently assembled around a subpopulation of mitochondria in the cell and subsequently cycled through distinct mitochondrial subpopulations (Supplementary Figs 3 and 4, and Supplementary Movies 1–6).

Given the persistent unidirectional cycling seen in some cells (Fig. 2a), we examined the directionality of cycling in the cell population. We found some cells exhibited persistent clockwise or anticlockwise cycling, although most commonly we observed a more stochastic result in which the depolymerization of actin filaments from one mitochondrial population was equally likely to be followed by repolymerization around adjacent mitochondria located to either side of the initial subpopulation. We also noted occasional apparent jumps across the cell to non-adjacent populations of mitochondria; however, analysis of time-lapse movies composed of *z*-stack max projections revealed that the observed actin jumping was in fact actin cycling onto intervening adjacent subpopulations of mitochondria either above or below the nucleus that were outside of the initial confocal plane. The cumulative sampling of mitochondrial subpopulations in a clockwise manner within a single cell is shown in the colour-coded max projection of the distribution of F-actin through time (Supplementary Fig. 3c). The average rate of actin cycling through the cell's total mitochondrial population is 14.0±0.8 min per cycle (mean±s.e.m.; *n*=16 cells), with a stepwise migration event every 3.7±0.3 min (mean±s.e.m.; *n*=16 cells).

Next, we asked whether the phenomenon of mitochondrial actin cycling was restricted to HeLa cells or could be seen in other cell types. We imaged LifeAct-GFP and Mito-DsRed2 in COS-7 cells (Supplementary Fig. 5 and Supplementary Movie 8) and noted very similar dynamics of actin assembly and disassembly around mitochondrial subpopulations within a cell, demonstrating that this process is not unique to HeLa cells. We also queried whether similar actin dynamics could be observed in primary cells by imaging actin and mitochondria over time in normal human epidermal keratinocytes (Supplementary Fig. 6 and Supplementary Movie 9). Again, we saw the periodic assembly and disassembly of actin filaments around mitochondrial subpopulations, indicating that this phenomenon is not related to long-term cell culture or transformation.

### Actin cycling is not regulated by membrane potential or ROS

**Figure 3:**
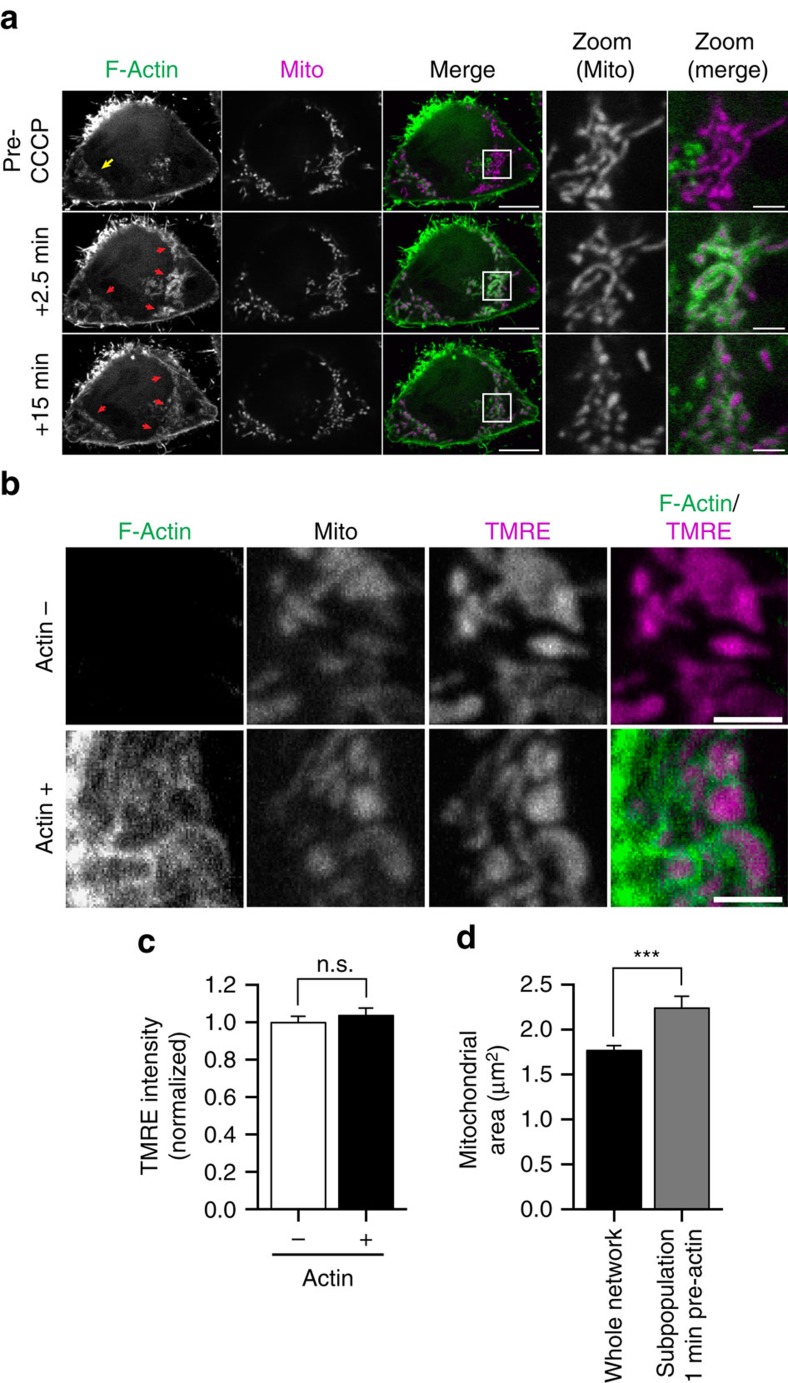
Actin cycles onto polarized and elongated mitochondria in healthy cells. (**a**) Time series demonstrating stable recruitment of F-actin (LifeAct-GFP) to mitochondria (Mito-DsRed2) in HeLa cells depolarized by treatment with 20 μM CCCP. Before CCCP treatment, cycling actin can be visualized on a subpopulation of HeLa cell mitochondria (yellow arrow, top panel). Within 2.5 min of CCCP treatment, actin was stabilized on >90% of all mitochondria (red arrows) and robust fission of all mitochondria was observed by 15 min post CCCP treatment. (**b**,**c**) Untreated cells expressing LifeAct-GFP and Mito-BFP display no significant difference in TMRE intensity between actin-positive and actin-negative mitochondria. (**d**) One minute before actin recruitment, mitochondria targeted for actin assembly are significantly larger than average mitochondria within the cell. Values represent means±s.e.m. ***P<0.001; NS, not significant. Scale bars, 10 μm (**a** full size); 2.5 μm (**a** zoom, **b**). Sample size: (**c**) 50 mitochondria from 10 cells per condition; (**d**, whole network) 27 cells; (**d**, actin-targeted subpopulation) 26 cells; *n*≥3 independent experiments in all cases.

Whole-cell depolarization of mitochondria induced by the mitochondrial uncoupler carbonyl-cyanide *m*-chlorophenyl-hydrazone (CCCP) has been reported to induce fission preceded by the rapid assembly of actin around mitochondria^13^. Consistently, we observed that within 2 min of CCCP addition, actin robustly assembled around the majority of mitochondria (Fig. 3a), effectively inhibiting further cycling of actin through mitochondrial subpopulations. CCCP-induced actin assembly on mitochondria was followed by robust mitochondrial fragmentation within 15 min of CCCP treatment (Fig. 3a).

These observations led us to ask whether the dynamic assembly of actin on specific mitochondrial subpopulations might occur in response to fluctuations in membrane potential. First, we compared actin localization to the intensity of TMRE (tetramethylrhodamine ethyl ester), a fluorescent dye that accumulates in the mitochondrial matrix of healthy, polarized mitochondria but does not remain sequestered in the matrix of depolarized mitochondria. We found that mitochondria were uniformly polarized within each HeLa cell, as has been observed in neurons^17^. We found no difference in the intensity of TMRE staining between the smaller, more circular mitochondria that were positive for actin, and the longer, more tubular mitochondria that showed no actin association (Figs 3b,c); further, we observed no cyclic alterations in TMRE intensity within a given cell over time that would correlate with the cyclic changes in actin ongoing in the cells. Thus, the association of actin filaments with subpopulations of cellular mitochondria is not correlated with fluctuations in their membrane potential.

Next, we asked whether actin cycling onto specific mitochondrial subpopulations occurred in response to production of reactive oxygen species (ROS). We induced localized ROS production within the mitochondrial matrix by expressing the mitochondrially targeted construct mito-KillerRed^18^ and then illuminating cells with 561 nm light (Supplementary Fig. 7a). Whole-cell illumination effectively bleached the KillerRed fluorescence, indicating activation of ROS production, but had no effect on the dynamic cycling of actin assembly/disassembly through mitochondrial subpopulations in time-lapse imaging for up to 30 min (Supplementary Fig. 7b).

Instead, the clearest predictor of actin cycling onto a mitochondrial subpopulation was mitochondrial size. When we examined the morphology of mitochondrial subpopulations one minute before actin assembly, we observed that prospective actin-positive mitochondria had an average area of 2.24±0.13 μm^2^ (mean±s.e.m.), significantly larger than the average size of all cellular mitochondria (1.77±0.05 μm^2^, mean±s.e.m., *P*<0.001; Fig. 3d).

### Actin cycling promotes localized mitochondrial fission

As the dynamics of mitochondrial fission and fusion play an important role in maintaining mitochondrial homeostasis, we investigated a possible role for actin cycling in regulating mitochondrial morphology. Based on our observations that actin preferentially cycles onto elongated mitochondria (Fig. 3d), and that actin-positive mitochondria are more fragmented (Fig. 1f–h and Supplementary Fig. 2b–d), we hypothesized that actin recruitment promotes rapid mitochondrial fission. To investigate whether actin recruitment precedes and possibly promotes mitochondrial fission events, we closely examined subregions of the cellular mitochondrial network before, during and after actin recruitment.

**Figure 4:**
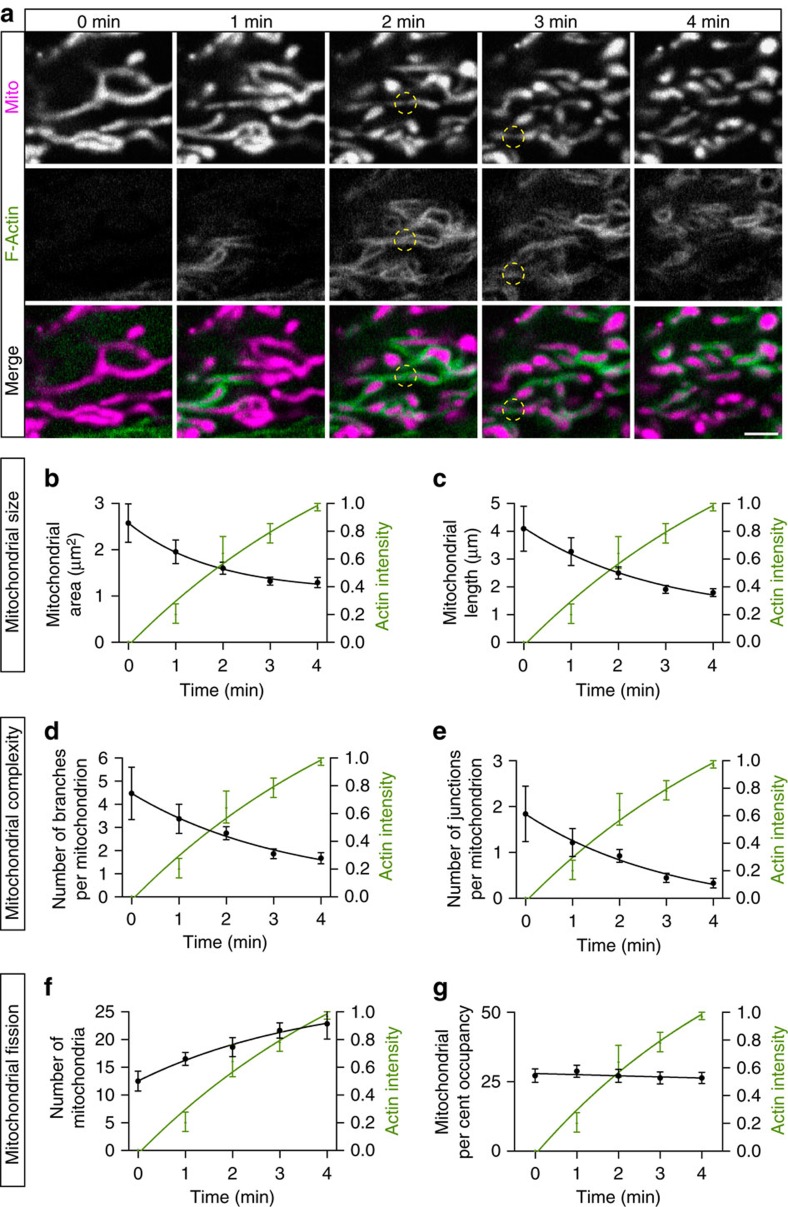
The mitochondrial network fragments in regions of focal actin assembly. (**a**) Confocal time series of F-actin (LifeAct-GFP) cycling onto elongated, tubular mitochondria (Mito-DsRed2) in a HeLa cell. Over 4 min, actin-positive mitochondria become increasingly fragmented. Yellow circles indicate sites of mitochondrial constriction. (**b**,**c**) Mitochondrial area and length decrease as actin intensity increases within a defined ROI (see Methods for details). (**d**,**e**) Actin recruitment to mitochondria is linked to changes in mitochondrial shape complexity, including decreased branches and junction points per mitochondrion. (**f**,**g**) Actin cycling onto mitochondria is associated with increased mitochondrial number, but no change in the overall mitochondrial per cent occupancy within the fixed ROI. Values represent mean±s.e.m. Scale bar, 2.5 μm (**a**). Sample size: (**b**–**g**) 6 cells; *n*=3 independent experiments.

Before actin recruitment, mitochondria were typically long and tubular (Fig. 4a). Following actin polymerization, mitochondria became constricted (specific sites indicated by yellow circles in Fig. 4a are noteworthy) and then fragmented into smaller pieces. Thus, actin recruitment to each mitochondrial subpopulation resulted in the localized fragmentation of mitochondrial networks (Supplementary Movie 10). In contrast, mitochondria that had not recruited actin within the same timeframe maintained their characteristic tubular and interconnected morphology.

We directly compared the kinetics of actin recruitment to changes in mitochondrial size (area and length) within specific regions of the cell (Fig. 4b,c). As actin intensity increased, the average individual mitochondrial size significantly decreased over a time scale of 4 min. Next, we examined the effects of localized actin assembly on the complexity of the mitochondria network. Over the same time scale and within the same localized region of the cell, we found that the increase in actin intensity was correlated with a decrease in the connectedness of the mitochondrial network, assessed as the number of branches per mitochondrion (Fig. 4d) and the number of junctions per mitochondrion (Fig. 4e; see Methods for details on the segmentation analysis used to assess these parameters).

We next assessed the frequency of mitochondrial fission in relation to actin association. The increased actin intensity, indicative of dynamic actin assembly around a subpopulation of cellular mitochondria, was closely correlated with a doubling in the number of mitochondria undergoing fission within this mitochondrial subpopulation (from 12.5±1.8 to 22.8±2.7; Fig. 4f). To demonstrate that these changes were due to remodelling of the resident mitochondrial population rather than movement of mitochondria into or out of the region of interest (ROI), we calculated the mitochondrial per cent occupancy for each ROI and found no significant change over time (Fig. 4g). Similarly, we tracked actin-associated mitochondrial fragmentation in *z*-stack three-dimensional projections (Supplementary Fig. 8), confirming that the observed fission is not due to the rotation or translocation of mitochondria in the *z*-axis.

Finally, to confirm that our actin markers were not contributing to the dynamic mitochondrial re-organization we observed upon actin recruitment, we performed live-cell imaging of cells expressing only Mito-DsRed2. Even in the absence of actin markers, we observed distinct subregions of the mitochondrial network undergoing fragmentation concurrent with continued elongation of the mitochondrial network throughout the rest of the cell (Supplementary Fig. 9). Thus, our live-imaging studies demonstrate that actin polymerization around mitochondrial subpopulations leads to a local enhancement of the frequency of fission, resulting in localized mitochondrial fragmentation and decreased interconnectivity.

### Actin polymerization inhibits mitochondrial fusion

Assembly of actin around mitochondrial subpopulations clearly enhances fission, but we wondered whether this assembly might also inhibit fusion. Specifically, the formation of F-actin networks around mitochondria might decrease organelle motility and serve as a barrier to fusion with neighbouring mitochondria. We found that, indeed, mitochondrial motility was significantly decreased when actin was assembled on mitochondria as compared with the motility of mitochondria without co-localized actin: we found the mean displacements over time for actin-positive and actin-negative mitochondria were 0.27±0.04 μm min^−1^ and 0.62±0.07 μm min^−1^, respectively (mean±s.e.m., *P*<0.001, *n*=16 cells per condition from 3 independent experiments).

**Figure 5:**
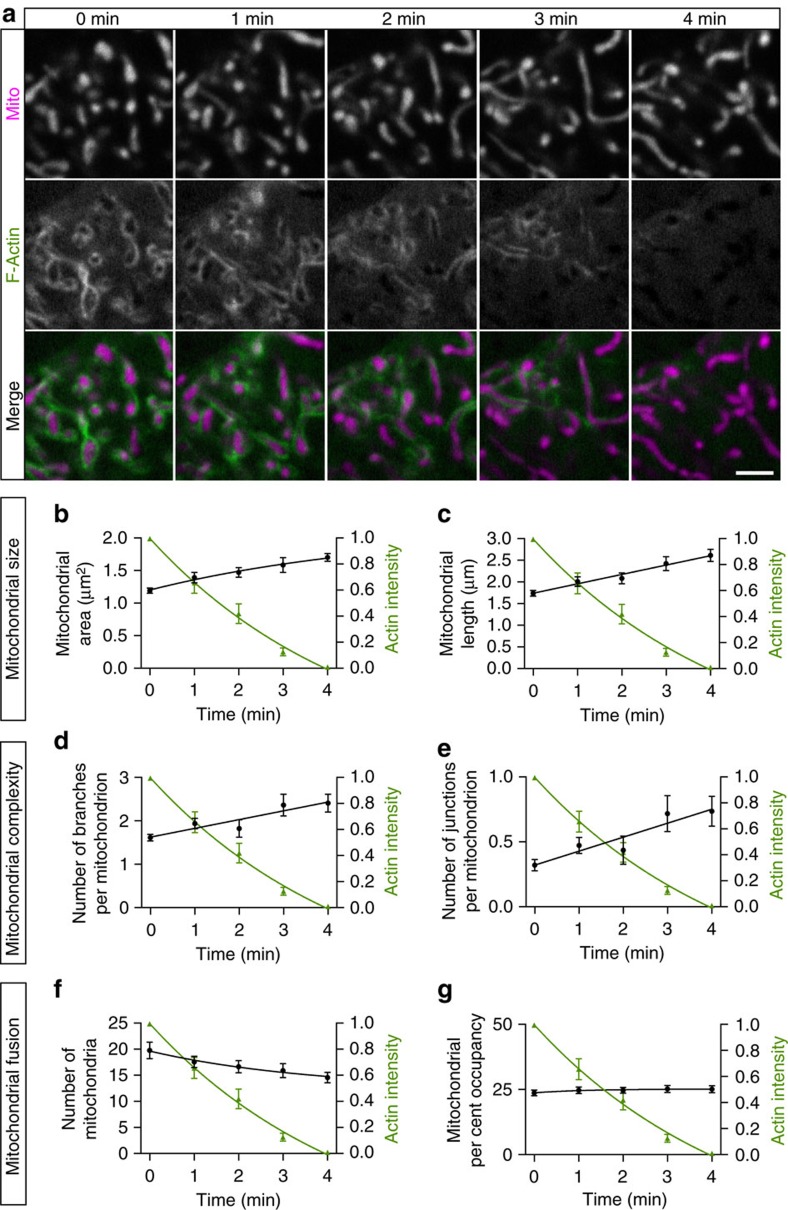
Mitochondria undergo rapid growth on actin disassembly. (**a**) Confocal time series showing actin (LifeAct-GFP) depolymerization from a subpopulation of fragmented, rounded mitochondria (Mito-DsRed2). On actin disassembly, mitochondria undergo rapid growth and fusion. (**b**,**c**) Mitochondrial area and length increase as actin intensity decreases. (**d**,**e**) Actin depolymerization augments mitochondrial complexity, resulting in an increased number of mitochondrial branches and junctions. (**f**,**g**) Actin disassembly leads to a decreased number of mitochondria, but has little effect on total mitochondrial percent occupancy, suggesting that fusion is enhanced on actin depolymerization. Values represent mean±s.e.m. Scale bar, 2.5 μm (**a**). Sample size: (**b**–**g**) 9 cells; *n*≥3 independent experiments.

Next, we asked whether actin disassembly was accompanied by an increase in the frequency of mitochondrial fusion. We focused on mitochondrial subpopulations enriched for actin and assayed for effects on mitochondrial morphology as the associated actin filaments disassembled (Fig. 5a). Quantitative analysis demonstrates that the decline in the fluorescence intensity of LifeAct-GFP over a 4 min time course of actin disassembly is correlated with increased mitochondrial area and length (Fig. 5b,c and Supplementary Movie 11). The interconnectedness of the mitochondrial network also increased as actin disassembled, as we noted significant increases in both the number of branches per mitochondrion and the number of junctions per mitochondrion (Fig. 5d,e). Finally, there was a decrease in the total number of individual mitochondria (Fig. 5f), but no change in the per cent occupancy within the examined ROI (Fig. 5g), consistent with an enhancement of fusion once actin disassembles from the given mitochondrial subpopulation. Tracking individual organelles, we observed that daughter mitochondria from fission events frequently moved in opposite directions before fusing with distinct, neighbouring mitochondria, thus promoting content mixing and network remodelling (Supplementary Fig. 10).

### Actin cycling requires Arp2/3 and formins

**Figure 6:**
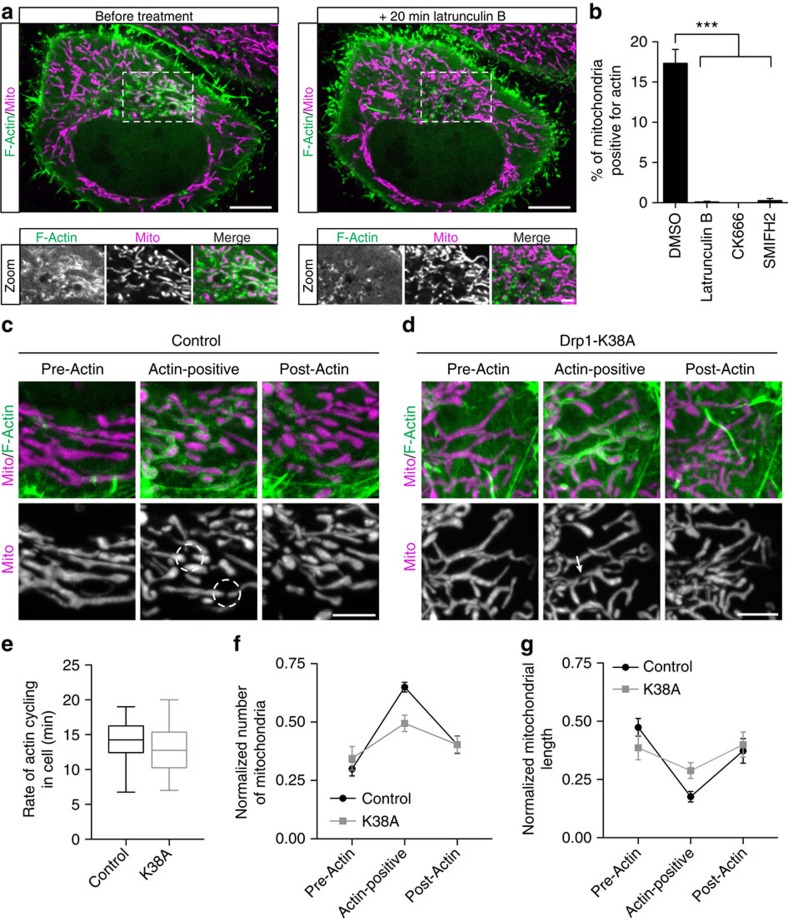
Actin cycling is dependent on actin polymerization by Arp2/3 and formins but not Drp1 activity. (**a**) Confocal images of F-actin (LifeAct-GFP) localized to a mitochondrial subpopulation (Mito-DsRed2) during constitutive cycling (left) in a HeLa cell. Actin recruitment to mitochondria was abolished on 20 min treatment with the actin depolymerizing drug Latrunculin B (2 μM) (right). (**b**) F-actin assembly on mitochondria was blocked in cells treated with 2 μM Latrunculin B (30 min), 84 μM CK-666 (1 h), or 25 μM SMIFH2 (1 h). (**c**,**d**) Maximum intensity projections, indicating F-actin (LifeAct-GFP) assembly onto and disassembly off of mitochondria (Mito-DsRed2) in HeLa cells transfected with either (**c**) an empty vector or (**d**) Drp1-K38A. For three-dimensional renderings of mitochondria undergoing fragmentation in control cells, see Supplementary Fig. 8. (**e**) Mean duration of actin cycling through the entire mitochondrial network in control and Drp1-K38A-expressing cells. (**f**,**g**) Normalized number and length of mitochondria before, during and after actin ssembly in either control or Drp1-K38A-expressing cells. Values represent mean±s.e.m. ***P<0.001. Scale bars, 10 μm (**a** full size); 5 μm (**c**,**d**); 2.5 μm (**a**, zoom). Sample size: (**b**, dimethyl sulfoxide, DMSO) 9 cells, (**b**, Latrunculin) 91 cells, (**b**, CK-666) 101 cells, (**b**, SMIFH2) 24 cells, (**e**) 16 cells, (**f**,**g** control) 15 cells, (**f**,**g**, K38A) 14 cells; *n*≥3 independent experiments in all cases.

The dynamics of actin cycling observed in live cells strongly suggest that polymerization of new, organelle-associated actin filaments is required. Therefore, this process should be inhibited by treatment with the actin-depolymerizing drug, Latrunculin B. Before treatment, we noted robust actin recruitment to a subset of mitochondria (Fig. 6a, left panel). After 20 min of Latrunculin B treatment, F-actin was not observed in association with any mitochondrial population in the cell (Fig. 6a,b).

Next, we asked whether the assembly of actin filaments onto mitochondria was nucleated by either Arp2/3 or formins. Following a 1 h treatment of HeLa cells with the Arp2/3 inhibitor CK-666, actin cycling on mitochondrial subpopulations was completely abolished and actin filaments were no longer associated with mitochondria (Fig. 6b). As recent work has implicated the formin INF2 and the formin-binding protein Spire 1C in mitochondrial fission^10^,^12^, we also tested whether formin activity is required for dynamic actin cycling. We treated cells with the small molecule formin inhibitor SMIFH2 for 1 h. Again, we noted the complete inhibition of dynamic actin cycling and the loss of actin association with mitochondria (Fig. 6b). Thus, both Arp2/3 and formins contribute to the dynamic formation of the mitochondria-associated actin filaments observed in our live-cell assays and inhibition of either nucleation mechanism is sufficient to block mitochondrial-associated actin cycling.

### Fission of actin-positive mitochondria requires Drp1

Actin recruitment to mitochondria leads to enhanced fission, as shown above. This led us to ask whether the observed fission of actin-positive mitochondria is dependent on the canonical fission machinery. We first investigated the localization of Drp1, the dynamin-related GTPase that regulates mitochondrial fission, during actin cycling. In HeLa cells transfected with GFP-Drp1, we did not observe preferential recruitment of Drp1 to mitochondria positive for actin (Drp1 puncta on actin-positive mitochondria 1.21±0.16 puncta per μm, Drp1 puncta on actin-negative mitochondria 1.23±0.20 puncta per μm (mean±s.e.m., *P*=0.93, *n*=11 cells from three independent experiments)). In fixed cells we observed comparable patterns of Drp1 staining on both phalloidin-positive and phalloidin-negative mitochondria, indicating that endogenous Drp1 is not preferentially recruited to actin-positive mitochondria.

Next, we asked whether Drp1 activity is necessary for actin-induced fission. To investigate this question, we transfected HeLa cells with Mito-DsRed2, LifeAct-GFP and either an empty vector control or Drp1-K38A, a dominant-negative mutant that has been demonstrated to inhibit Drp1 activity^19^. Consistent with previous reports, expression of Drp1-K38A resulted in profound mitochondrial elongation and hyperfusion. In both control and Drp1-K38A-expressing cells, we identified actin assembly onto and disassembly off of subpopulations of mitochondria (Fig. 6c,d). Further, we found that expression of the dominant-negative Drp1 construct did not affect the mean time required for actin to cycle onto all mitochondrial subpopulations (Fig. 6e). However, mitochondria in cells expressing Drp1-K38A failed to undergo robust mitochondrial fission on actin assembly. Quantitative analysis of *z*-stack projections indicates a significant increase in mitochondrial fission on actin recruitment to mitochondria in control cells, but only a modest change in mitochondrial number on actin recruitment to mitochondria in Drp1-K38A-expressing cells (Fig. 6f). Similarly, we identified a 63% decrease in mitochondrial length on actin recruitment in control cells and only a 25% decrease in cells transfected with Drp1-K38A (Fig. 6g). Consistent with this observation, cells treated with the Drp1 inhibitor^20^ Mdivi-1 did not display robust mitochondrial fission on actin assembly (Supplementary Fig. 11). Therefore, actin cycles through mitochondrial subpopulations independently of Drp1 activity, but Drp1 is required for efficient fragmentation and remodelling of the mitochondrial network following localized actin assembly.

### Actin assembles at ER–mitochondria contacts

**Figure 7:**
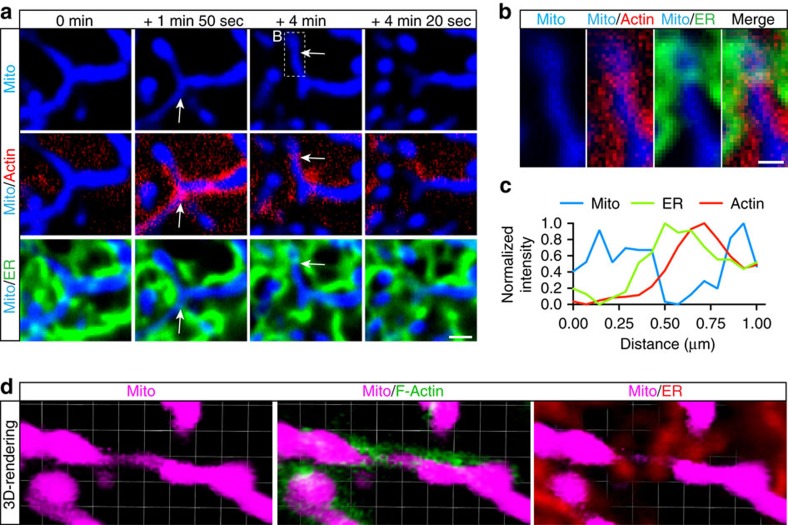
Actin assembles at ER–mitochondria contact sites. (**a**) Confocal image of GFP-actin (red) recruitment to elongated, Mito-SNAP-labelled mitochondria (blue) in a HeLa cell. Actin assembly is enriched at sites of ER tubule (DsRed2-ER, green) overlap with mitochondria. Arrows indicate regions of Actin/ER co-localization at prospective sites of mitochondrial fission. (**b**) Enlarged image of Box B (+4 min), demonstrating co-localization of actin and ER at the site of mitochondrial fission. (**c**) Line scan indicating overlapping peak actin and ER intensity at site of mitochondrial constriction in (**b**). (**d**) Three-dimensional rendering of F-actin (LifeAct-GFP) and ER-tubules (DsRed2-ER) assembled on a constricted HeLa cell mitochondrion (Mito-SNAP). Scale bars, 1 μm (**a**), 0.5 μm (**b**) and 0.75 μm per unit (**d**).

Recent work has identified key roles for ER-mitochondrial contact sites in lipid exchange, calcium signalling as well as the initiation of mitochondrial fission^9^,^21^. Thus, we investigated whether actin preferentially cycles onto these inter-organelle junctions. Before actin recruitment, we frequently observed the ER in the vicinity of mitochondria (Fig. 7a). As actin polymerized on elongated mitochondria, we identified uniform actin recruitment to the outer mitochondrial membrane with specific enrichment of actin at sites of ER–mitochondria overlap (Fig. 7a,b). Line-scan analysis indicated close apposition of actin with ER at sites of mitochondrial constriction before fission (Fig. 7b,c). In three-dimensional renderings of these ER–mitochondria contact sites, we see actin specifically enriched at points of mitochondrial constriction, where we observe the initiation of a fission event (Fig. 7d). Thus, dynamic actin cycling may facilitate mitochondrial fission through the induction of ER-dependent mitochondrial constriction. This interpretation is consistent with recent work demonstrating a role for the mitochondrially localized Spire 1C and ER-localized INF2 in the formation of mitochondrial constrictions^10^,^12^.

### Effects of actin cycling on the mitochondrial network

**Figure 8:**
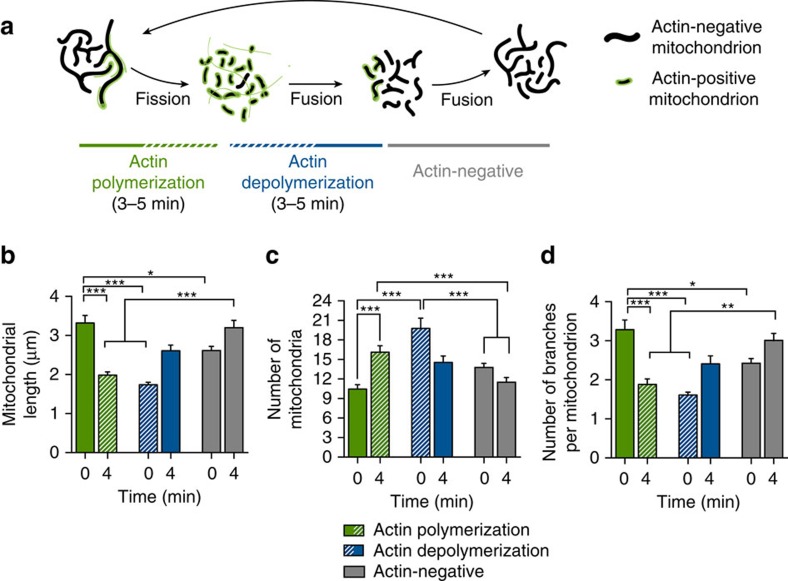
Actin cycling through mitochondrial subpopulations regulates steady-state mitochondrial morphology. (**a**) Model: actin polymerizes on a subpopulation of elongated mitochondria, inducing robust fragmentation. After ∼3–5 min, actin then depolymerizes, allowing the fragmented mitochondria to refuse and slowly increase in size and complexity. Actin-negative mitochondria slowly continue to grow until they become locally hyperfused, at which point actin is recruited and the cycle repeats. (**b**) Mitochondrial size decreases with actin recruitment and recovers on subsequent actin depolymerization. (**c**) Actin polymerization induces rapid mitochondrial fission, whereas depolymerization promotes slow fusion. (**d**) Mitochondrial complexity decreases with actin recruitment and recovers on actin depolymerization. Values represent mean±s.e.m. ***P<0.001, **P<0.01 and *P<0.05. Sample size: (**b**–**d**, actin polymerization) 26 cells, (**b**–**d**, actin depolymerization) 9 cells, (**b**–**d**, actin negative) 32 cells; *n*≥3 independent experiments in all cases.

Our data fit a model in which the active polymerization of actin on a mitochondrial subpopulation promotes fission, whereas actin disassembly allows fusion, locally restoring the integrity of the mitochondrial network (Fig. 8a). To quantitatively test this model, we directly compared mitochondrial length, number of branches and overall mitochondrial number in subcellular regions of comparable size: (1) before and after actin polymerization, (2) before and after actin depolymerization and (3) in cellular regions in which no actin cycling was observed over a 4 min time period. As shown in Fig. 8b,c, active polymerization of actin led to a significant decrease in mitochondrial length within 4 min, with a corresponding increase in mitochondrial number. In contrast, actin depolymerization was followed by an increase in mitochondrial length and a decrease in mitochondrial number over a similar 4 min time course. Of note, in the absence of associated actin filaments, we noted a steady growth in mitochondrial length over the same 4 min window and a small decrease in the number of mitochondria, suggesting that in the absence of external factors such as actin dynamics, fusion may dominate over fission.

To assess the effects of actin assembly/disassembly on the interconnectedness of the mitochondrial network, we measured the average number of branches per mitochondrion over time (Fig. 8d). Again, actin polymerization led to a significant fragmentation of the network, which was reversed upon actin depolymerization. As with mitochondrial length, we saw that in the absence of associated actin filaments, there is a tendency for the mitochondrial network to increase in complexity over time.

## Discussion

In this study, we used live-cell imaging to examine actin dynamics during mitochondrial fission and fusion. We propose that cyclic actin assembly/disassembly on mitochondrial subpopulations acts to regulate steady-state mitochondrial morphology. Actin cycles through mitochondrial subpopulations in the cell, assembling on the outer membrane of elongated mitochondria to promote fission events and transiently inhibit mitochondrial fusion and motility. Subsequent disassembly of actin facilitates reconfiguration of the mitochondrial network and mixing of mitochondrial contents. Cycles of actin assembly and disassembly survey and regulate the morphology of the total mitochondrial population within the cell in 14 min. In contrast to the chiral symmetry that the actin cytoskeleton can adopt in developing fibroblasts^22^, we found that actin showed no directional bias in its recruitment to mitochondrial subpopulations. Further, we found no evidence for fluctuations in mitochondria membrane potential or ROS production that might locally promote actin assembly around mitochondrial subpopulations.

At any point in time, actin filaments are associated with ∼20% of cellular mitochondria. This association is transient, as actin remains assembled on mitochondrial subpopulations for 3–5 min. During this period, individual organelles exhibit enhanced fission and suppressed fusion, leading to localized fragmentation of the mitochondrial network. Consistent with previous reports^9^,^12^, we frequently observe fission events in the vicinity of mitochondria–ER contacts.

Fragmentation of actin-positive mitochondria is dependent on Drp1 activity, as inhibition of Drp1 by either treatment with the inhibitor Mdivi-1 or expression of a dominant-negative Drp1-K38A blocks the local remodelling of actin-positive mitochondria. Of note, inhibition of Drp1 has no effect on the rate of actin cycling through the mitochondrial network, indicating that actin assembly and disassembly from mitochondrial subpopulations is not dependent on successful mitochondrial fission. Using both fixed and live-cell techniques, we observed no differences in the localization or density of Drp1 puncta between actin-negative and actin-positive mitochondria. However, Drp1 puncta on actin-positive mitochondria may be differentially primed for fission through posttranslational modifications^23^,^24^, association with outer-membrane fission receptors such as Mff or Fis1 or even simply through binding to actin itself^8^,^14^.

Actin assembly on mitochondria results in a ∼50% reduction in mitochondrial motility. This reduction in motility may contribute to the inhibition of mitochondrial fusion during local network remodelling. Specifically, decreased mitochondrial velocity reduces the probability of inter-mitochondrial contact events, which are necessary for successful fusion^25^. In addition, the formation of an actin cage around mitochondria may obstruct the association of mitofusin proteins between adjacent mitochondria, further potentiating the fusion block. On actin disassembly from a mitochondrial subpopulation, the organelles rapidly undergo fusion to mix their contents and restore a tubular mitochondrial network.

We found that F-actin recruitment to mitochondria is dependent on both Arp2/3 and formins. Curiously, inhibition of either actin-filament nucleator entirely abolished actin cycling through mitochondrial subpopulations, suggesting that Arp2/3 and formin family proteins work together to facilitate actin polymerization on the mitochondrial outer membrane. Work in budding yeast has identified Arp2/3-dependent actin clouds around fragmenting mitochondria^26^. In addition, Arp2/3 has been identified on the mitochondrial outer membrane of mammalian cells, where it regulates mitochondrial morphology^13^. Recently, the formin-binding protein Spire1C was identified on the mitochondria outer membrane, where it works in close association with the ER-localized formin INF2 to regulate mitochondrial length^12^. Actin depolymerization by Latrunculin B, as well as depletion of Arp2/3 or certain formins results in robust mitochondrial elongation^10^,^12^,^13^. Moving forward, it will be interesting to examine the signalling pathways responsible for activation of actin-nucleating proteins on elongated mitochondria, leading to localized filament assembly, as well as to investigate actin filament organization at higher resolution.

We propose that actin cycling through mitochondrial subpopulations serves as a surveillance method to identify and fragment regions of elongated mitochondria. This hypothesis is consistent with previous observations indicating that elongated mitochondria are more likely to undergo fission^25^. Regional regulation of mitochondrial dynamics may confer a number of advantages in the maintenance of mitochondrial networks. First, localized mitochondrial network remodelling could be important for mitochondrial biogenesis and the maintenance and distribution of mitochondrial DNA nucleoids^27^,^28^. Second, mitochondrial network remodelling may facilitate efficient transport of organelles through the cytoplasm, as previous work from our lab has demonstrated that interactions between organelles can restrict organelle trafficking^29^. An intricately connected mitochondrial network might have the same effect, such that local remodelling would be permissive for intracellular trafficking. Third, local remodelling of mitochondrial subpopulations allows for the fragmentation of individual, overgrown mitochondria without substantially altering the integrity of the mitochondrial network as a whole. As network-wide mitochondrial elongation has been linked to cellular senescence^30^, actin cycling may be a highly important homeostatic function to offset premature senescence. Fourth, actin polymerization onto only one mitochondrial subpopulation at a time ensures that the cellular actin pool is not depleted, consistent with observations that most dynamic cellular actin is enriched at the cortex rather than in close association with mitochondria. Finally, constitutive actin cycling may function as a quality-control mechanism to isolate damaged or depolarized mitochondria, which are less able to re-fuse with other healthy organelles^31^, for degradation by autophagy, again consistent with a homeostatic role for this mechanism.

Constitutive actin cycling may be efficiently tuned to maintain mean mitochondrial size, complexity and number over each 14 min cycle, while also maintaining heterogeneity in the morphology of individual mitochondria. During mitosis, cells in prophase and metaphase were found to have both more fragmented mitochondria and actin around their mitochondria^13^. Thus, actin cycling may be upregulated in mitotic cells whose mitochondrial network must efficiently fragment in preparation for segregation into daughter cells^32^,^33^,^34^. Actin cycling may also regulate mitochondrial morphology in postmitotic cells such as neurons, which require precise regulation of mitochondrial dynamics and quality control for their viability^35^,^36^.

In summary, we propose a model in which mitochondrial network morphology is not regulated by a dynamic equilibrium between fission and fusion at the level of the individual organelle, but rather at a network level by the actin cytoskeleton. Mitochondria within the cell constantly grow and fuse, generating an increasingly branched and interconnected network. This steady growth is counterbalanced by intervals of fast and spatially restricted actin-dependent fission events, primarily at ER-mitochondrial contact sites. Although we focused primarily on the HeLa model system, we note similar cycles of actin assembly/disassembly regulating mitochondrial fission and fusion in another cell line, as well as in primary human cells, suggesting that actin-dependent cyclic mitochondrial fission may be a conserved homeostatic mechanism that functions to regulate the cellular mitochondrial network.

## Methods

### Reagents

Constructs used include: EGFP-actin (Clontech), LifeAct-EGFP, F-tractin-EGFP (Addgene), mCherry-LifeAct (Dominguez Lab, University of Pennsylvania), Mito-DsRed2 (gift from T. Schwarz, Harvard Medical School, Boston, MA) recloned into pSBFP2-C1 (Addgene) and SNAP-Tag (NEB), pKillerRed-dMito (Evrogen), Drp1 (Addgene) recloned into pEGFP (Clontech), Drp1-K38A (Addgene), DsRed2-ER (gift from A. Akhmanova, Utrecht University, Utrecht, The Netherlands).

### Cell culture and transfections

HeLa-M cells (A. Peden, Cambridge Institute for Medical Research, Cambridge, UK) and COS-7 cells (ATCC) were cultured in DMEM medium (10-027-CV, Corning) supplemented with 10% fetal bovine serum (vol/vol) and 1% glutamax, and maintained at 37 °C in a 5% CO_2_ incubator. Normal human epidermal keratinocytes were isolated from de-identified discarded neonatal human foreskin, which was incubated for 12 h at 4C in 2.4 U ml^−1^ dispase II^37^. Sterile forceps were used to separate the underlying dermis. The epidermal sheet was transferred to a 60 mm tissue culture plate and incubated in 0.25% Trypsin for 10 min at 37C, then neutralized with 1 ml fetal bovine serum. Sterile forceps were used to scrape the epidermal sheet against the dish to dissociate cells. The suspension was passed through a 40 μm strainer, then centrifuged at 200 g for 5 min. The cell pellet was re-suspended in 5 ml medium M154 (Invitrogen) containing 0.07 mM calcium. Normal human epidermal keratinocytes were passaged up to six times. All cells were seeded in 35 mm glass-bottom dishes and transfected ∼24 h before imaging using FuGENE 6 (Promega). Cells were treated with 2 μM Latrunculin B for 30 min (BML-T110-0001, Enzo), 25 μM SMIFH2 for 1 h (S4826, Sigma), 84 μM CK-666 for 1 h (SML0006, Sigma) or 50 μM Mdivi-1 for 16 h (M0199, Sigma) before imaging. CCCP (Sigma-Aldrich) was given at 20 μM. Cells were incubated in 30 nM TMRE in complete DMEM (T-669, Molecular Probes) for 15 min and washed 2 × in DMEM before live-cell imaging. Cells expressing Mito-SNAP were incubated with 2.5 μM SNAP-cell 647-SiR (S9102S, NEB) for 30 min and washed 2 × before imaging.

### Live-cell imaging and analysis

All images were acquired on a spinning-disk confocal (UltraVIEW VoX; Perkin Elmer) on a Nikon Eclipse Ti microscope using an Apochromat × 100 1.49 numerical aperture oil-immersion objective (Nikon) in a temperature-controlled chamber (37 °C). Digital images were acquired with an EM charge-coupled device camera (C9100; Hamamatsu Photonics) using Volocity software (PerkinElmer) at 1 frame every 1–30 s or 1 frame per minute. Specifically, after the plates are mounted on the microscope, we selected the first observed cell with identifiable actin recruitment to mitochondria. The cell was imaged for no longer than 15–20 min with <10% laser power and then immediately a second cell was imaged in a separate area of the plate. No more than four cells were imaged from a single plate. Cells were excluded from analysis if they displayed signs of phototoxicity such as blebbing or vacuolization. All experiments were performed on at least three independent occasions. Time-lapse maximum intensity projection movies were created by acquiring nine *z*-slices separated by 0.5 μm step size every 30 s for 15–20 min. To induce ROS production, we photobleached a region or the entire HeLa cell expressing pKillerRed-dMito with a 561-nm laser at 100% for 100 iterations.

Images of mitochondria (both confocal slices and maximum intensity projections) were segmented using the pixel classification feature of the interactive learning and segmentation toolkit (Ilastik 1.1.5) and analysed by Fiji (NIH). Segmentation of TOM20 labelled mitochondria was carried out using Volocity (PerkinElmer). Automated image analysis was manually verified by comparing the segmentation mask to the original image files. Measures of mitochondrial area, number, circularity and fractional occupancy were generated using the ‘analyse particles' function in Fiji (NIH) with a minimum area of 0.25 μm^2^. Measures of mitochondrial length, junctions (voxels with three or more neighbors) and branches (slab segments connecting end points to either junctions or other endpoints) were determined using the ‘skeletonize' and ‘analyse skeleton' plugins in Fiji (NIH). Analyses were carried out on either whole cells or square ∼100 μm^2^ ROIs around subpopulations of actin-positive mitochondria.

For actin recruitment analyses in Figs 4 and 8, time 0 is defined as 1 min before the first observable actin recruitment to >25% of mitochondria within the ROI. For actin disassembly analyses in Figs 5 and 8, time 4 is defined as 1 min subsequent to the disassembly of actin from >75% of mitochondria within the ROI. For Fig. 7, segmentation of mitochondria from both control and Drp1-K38A cells was carried out on maximum intensity projections and mitochondrial length and number was normalized per cell. All other analyses were performed on single plane images. Line scans were generated using ImageJ (NIH) and normalized per fluorescent tag. Three-dimensional renderings were generated using Volocity (PerkinElmer). All images were assembled using Fiji (NIH) and Illustrator (Adobe). Statistics and graphing were performed using Prism (GraphPad) software. Comparisons of two data sets were performed using unpaired two-tailed Student's *t*-test for normally distributed data sets and Mann–Whitney test for non-normally distributed data sets. Comparisons for multiple data sets were performed using one-way analysis of variance with Tukey's *post-hoc* test for normally distributed data sets and Kruskal–Wallis test with Dunn's multiple comparison test for non-normally distributed data sets.

### Immunofluorescence

HeLa cells seeded on 35 mm glass-bottom dishes were fixed in 3.7% paraformaldehyde at 37 °C for 12 min, washed in PBS × 3 and permeabilized in 0.2% Triton X-100 in PBS for no longer than 5 min at room temperature. Cells were again washed in PBS × 3 and blocked in 0.5% BSA in PBS for 10 min at room temperature. Cells were then incubated for 30 min with Phalloidin-488 (1:200; A12379, ThermoFisher), anti-Tom20 rabbit polyclonal antibody (1:50; sc-11415, Santa Cruz) and/or anti-DRP1 mouse monoclonal antibody (1:100; ab56788, Abcam) in PBS with 0.5% BSA. Cells were then washed in PBS × 3 and incubated with either AlexaFluor goat-anti-rabbit-594 (1:300; A-11037, ThermoFisher) or AlexaFluor goat-anti-rabbit 555 (1:300; A21429, ThermoFisher), AlexaFluor goat-anti-mouse-647 (1:300; A21052, ThermoFisher) and Hoechst dye (62249; ThermoFisher). Cells were washed in PBS × 3 and imaged.

### Data availability

The data that support the findings of this study are available from the corresponding author upon request.

## Additional information

**How to cite this article:** Moore, A. S. *et al*. Dynamic actin cycling through mitochondrial subpopulations locally regulates the fission–fusion balance within mitochondrial networks. *Nat. Commun.* 7:12886 doi: 10.1038/ncomms12886 (2016).

## Supplementary Material

Supplementary Information Supplementary Figures 1 - 11

Supplementary Movie 1 F-actin cycles through mitochondrial subpopulations. Live cell confocal movie of HeLa cell expressing LifeAct-GFP (green) and Mito-DsRed2 (magenta). Over 15 min, F-actin assembles onto and disassembles off of distinct subsets of mitochondria, cycling through the entire mitochondrial network in a persistent clockwise direction. White arrow indicates leading edge of mitochondria-localized actin. Movie corresponds to Fig. 2. Images were captured at 1 frame per 30 sec. Time min:sec. Scale bar, 10 μm.

Supplementary Movie 2 LifeAct-GFP cycles through mitochondrial subpopulations (example 2). Live cell confocal movie of HeLa cell expressing LifeAct-GFP (green) and Mito-DsRed2 (magenta). Over 17 min, LifeAct cycles through all mitochondrial subpopulations. White arrow indicates leading edge of mitochondria-localized actin. Movie corresponds to Supplementary Fig. 1. Images were captured at 1 frame per 15 sec. Time min:sec. Scale bar, 10 μm.

Supplementary Movie 3 Maximum intensity projection of LifeAct-GFP cycling through mitochondrial subpopulations. Live cell movie of maximum intensity projections of HeLa cell expressing LifeAct-GFP (green) and Mito-DsRed2 (magenta). Actin cycles through >90% of mitochondria over 15 min. White arrows indicate actin-positive mitochondria. Maximum intensity projections are composed of 9x0.5μm confocal slices. Images were captured at 1 frame per 30 sec. Time min:sec. Scale bar, 10 μm.

Supplementary Movie 4 3D projection of LifeAct-GFP cycling through mitochondrial subpopulations. Live cell 3D movie of LifeAct-GFP (green) cycling onto mitochondria (magenta) in a HeLa cell. Actin polymerization on mitochondria dramatically reorganizes local mitochondrial network morphology. 3D renderings were generated from confocal stacks consisting of 9x 0.5μm confocal slices. Images were captured at 1 frame per 30 sec. Time min:sec. Scale bar, 10μm.

Supplementary Movie 5 F-actin cycling is visible in the majority of cells. Live cell confocal movie of multiple HeLa cells expressing LifeAct-GFP (green) and Mito-DsRed2 (magenta). Over time, F-actin cycles through subpopulations of mitochondria in all of the cells in the window. White arrows indicate actin-positive mitochondrial. Images were captured at 1 frame per 5 sec and displayed at 1 frame per 30 sec. Time min:sec. Scale bar, 10 μm.

Supplementary Movie 6 GFP-actin cycles through mitochondrial subpopulations. Live cell confocal movie of HeLa cell expressing GFP-actin (green) and Mito-DsRed2 (magneta). GFP-actin enriches on distinct subpopulations of mitochondria, effectively cycling through the entire mitochondrial network over 15 min. White arrow indicates actin-positive mitochondria. Movie corresponds to Supplementary Fig. 2. Images were captured at 1 frame per 15 sec. Time min:sec. Scale bar, 10 μm.

Supplementary Movie 7 F-Tractin-GFP cycles through mitochondrial subpopulations. Live cell confocal movie of HeLa cell expressing F-Tractin-GFP (green) and Mito-DsRed2 (magenta). Over 10 min, F-Tractin cycles through distinct subpopulations of mitochondria in the cell. White arrow indicates actin-positive mitochondria. Images were captured at 1 frame per 10 sec. Time min:sec. Scale bar, 10 μm.

Supplementary Movie 8 F-actin cycles through mitochondrial subpopulations in Cos-7 cells. Live cell confocal movie of Cos-7 cell expressing LifeAct-GFP (green) and Mito-DsRed2 (magenta). Over 16 min, F-actin cycles through subpopulations of mitochondria in the cell. White arrows indicate actin-positive mitochondria. Movie corresponds to Supplementary Fig. 3. Images were captured at 1 frame per 5 sec. Time min:sec. Scale bar, 10 μm.

Supplementary Movie 9 F-actin cycles through mitochondrial subpopulations in normal human epidermal keratinocytes. Live cell confocal movie of normal human epidermal keratinocytes expressing LifeAct-GFP (green) and Mito-DsRed2 (magenta). F-actin cycles through mitochondrial subpopulations over ~13 min. Yellow arrow indicates actin-positive mitochondria. Movie corresponds to Supplementary Fig. 4. Images were captured at 1 frame per 10 sec. Time min:sec. Scale bar, 10 μm.

Supplementary Movie 10 F-actin cycling through a mitochondrial subpopulation promotes mitochondrial fission and fragmentation. Live cell confocal movie of actin cycling onto mitochondria in a HeLa cell expressing LifeAct-GFP (green) and Mito-DsRed2 (magenta). Actin gradually polymerizes on the outer surface of branched, elongated mitochondria. Over 3-5 min, actin assembly promotes robust mitochondrial fragmentation. Movie corresponds to Fig. 4. Images were captured at 1 frame per 5 sec. Time min:sec. Scale bar, 5 μm.

Supplementary Movie 11 F-actin disassembly promotes mitochondrial fusion. Live cell confocal movie of actin cycling off of mitochondria in a HeLa cell expressing LifeAct-GFP (green) and Mito-DsRed2 (magenta). Over 3-5 min, actin disassembles from fragmented mitochondria, allowing mitochondria to rapidly restore their tubular morphology. Movies correspond to Fig. 5. Images were captured at 1 frame per 15 sec. Time min:sec. Scale bar, 5 μm.
