# Supplementary material for: Dynamic actin cycling through mitochondrial subpopulations locally regulates the fission–fusion balance within mitochondrial networks
Source: Nat Commun. 2016 Sep 30;7:12886. doi: 10.1038/ncomms12886 (PMC5056443; doi:10.1038/ncomms12886)
Supplement: Supplementary Information — Supplementary Figures 1 - 11 [file ncomms12886-s1.pdf]

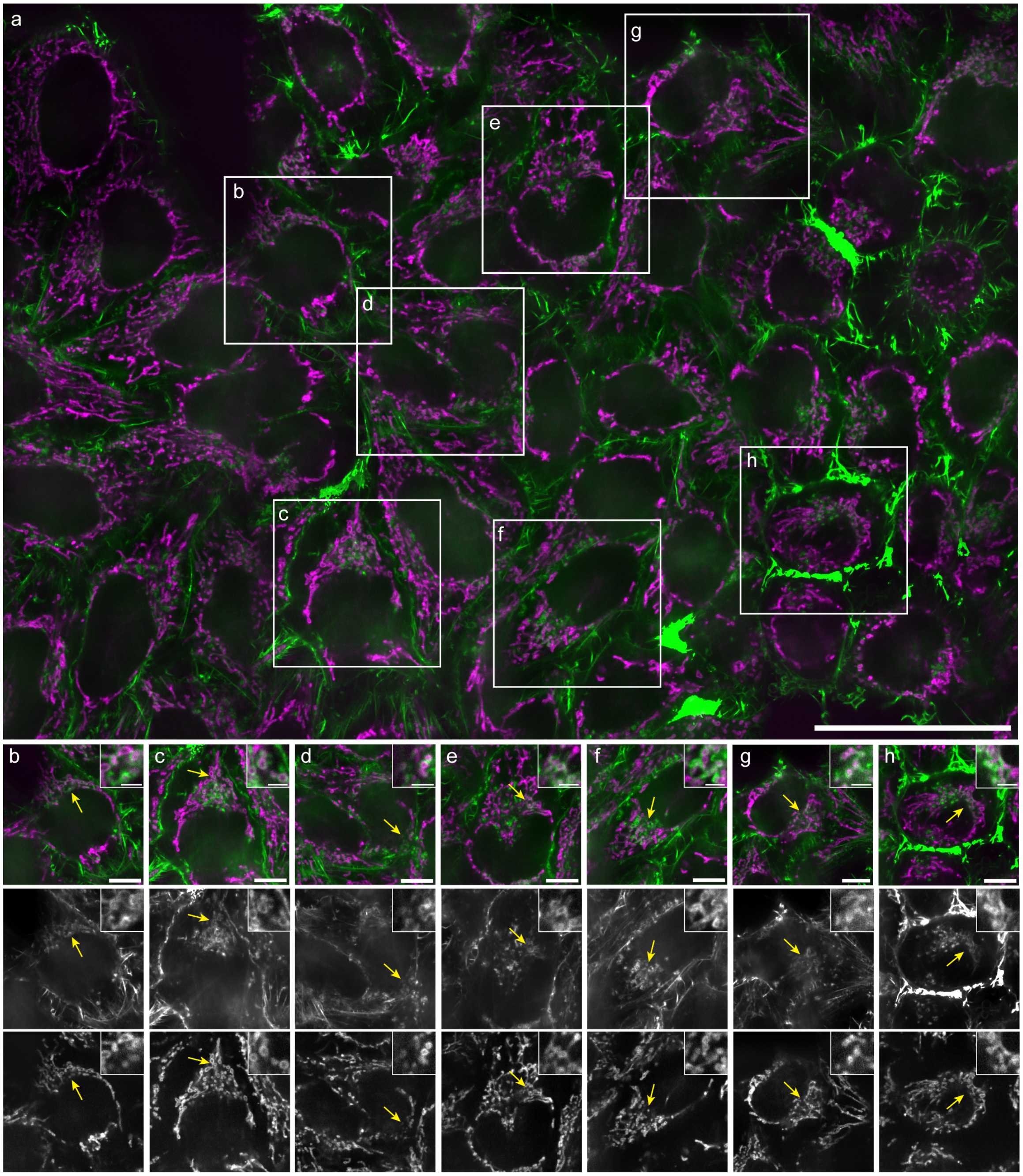


**Supplementary Figure 1. F-actin localization to subsets of mitochondria is observed in the majority of HeLa cells.** (**a**) Stitched confocal image of a ~260x190 µm confluent region of fixed HeLa cells stained with anti-TOM20 antibody (magenta) and phalloidin (green). (**b-h**) Enlarged images of randomly selected HeLa cells from the field shown in (a). Yellow arrows indicate actin-positive regions displayed in enlarged insets. Images within the insets were further contrast enhanced for clarity. Scale bars (a), 50 µm; (b-h), 10 µm; (b-h insets), 2.5 µm.

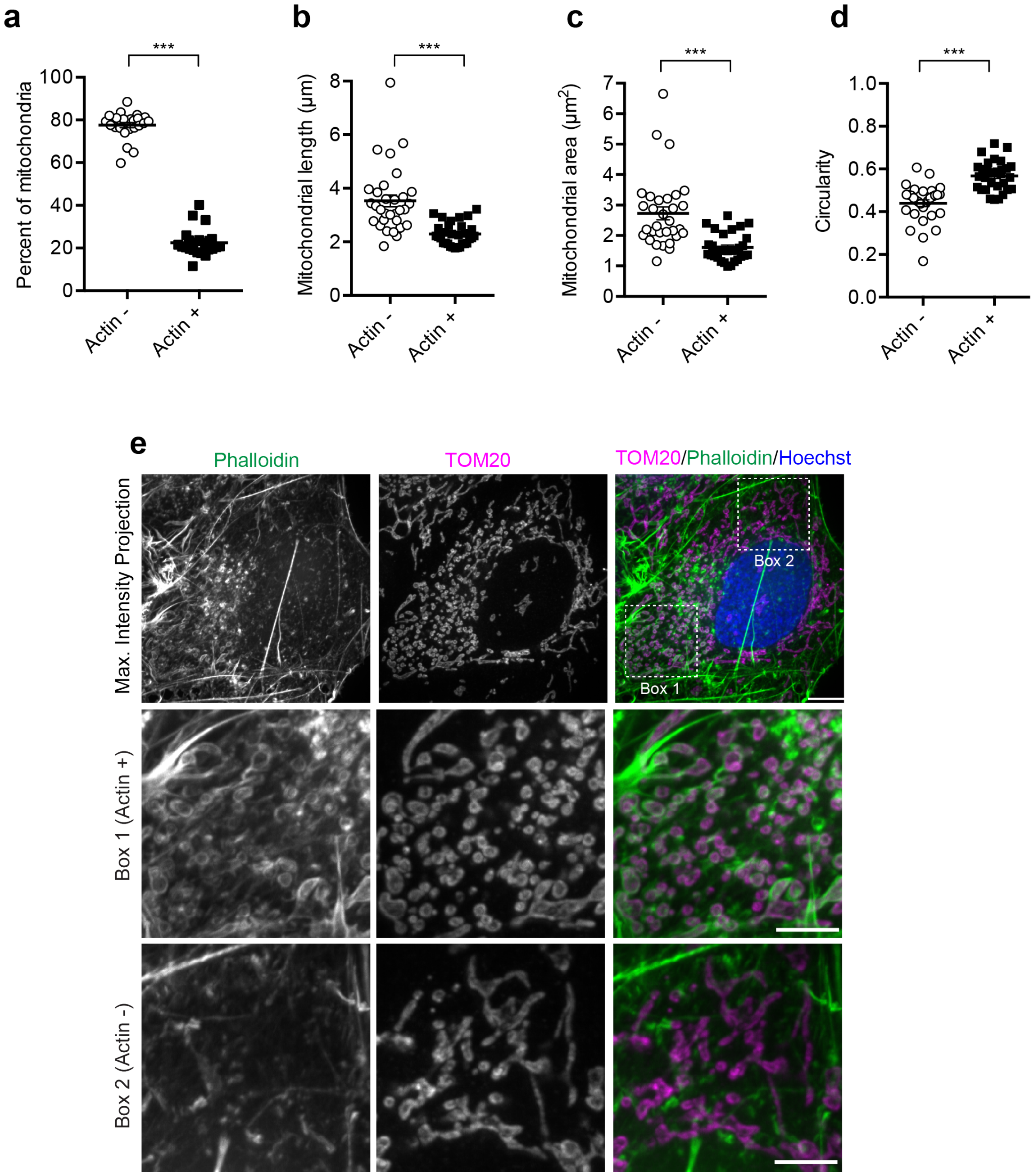


**Supplementary Figure 2. Phalloidin localizes to a subset of mitochondria within each HeLa cell**

(**a**) Percentage of Phalloidin-positive mitochondria per cell. (**b-d**) Quantifications of length, area, and circularity for actin-negative and actin-positive mitochondria. (**e**) Maximum intensity projection (4 z-slices separated by 1 µm intervals) of cell displayed in figure 1i. Error bars represent mean ± S.E.M. *** $p$ <0.001. Scale bars (**e** full size), 10 µm; (**e** zoom), 5 µm. Sample size (a), 24 cells; (b-d), 31 cells; N≥3 independent experiments in all cases.g

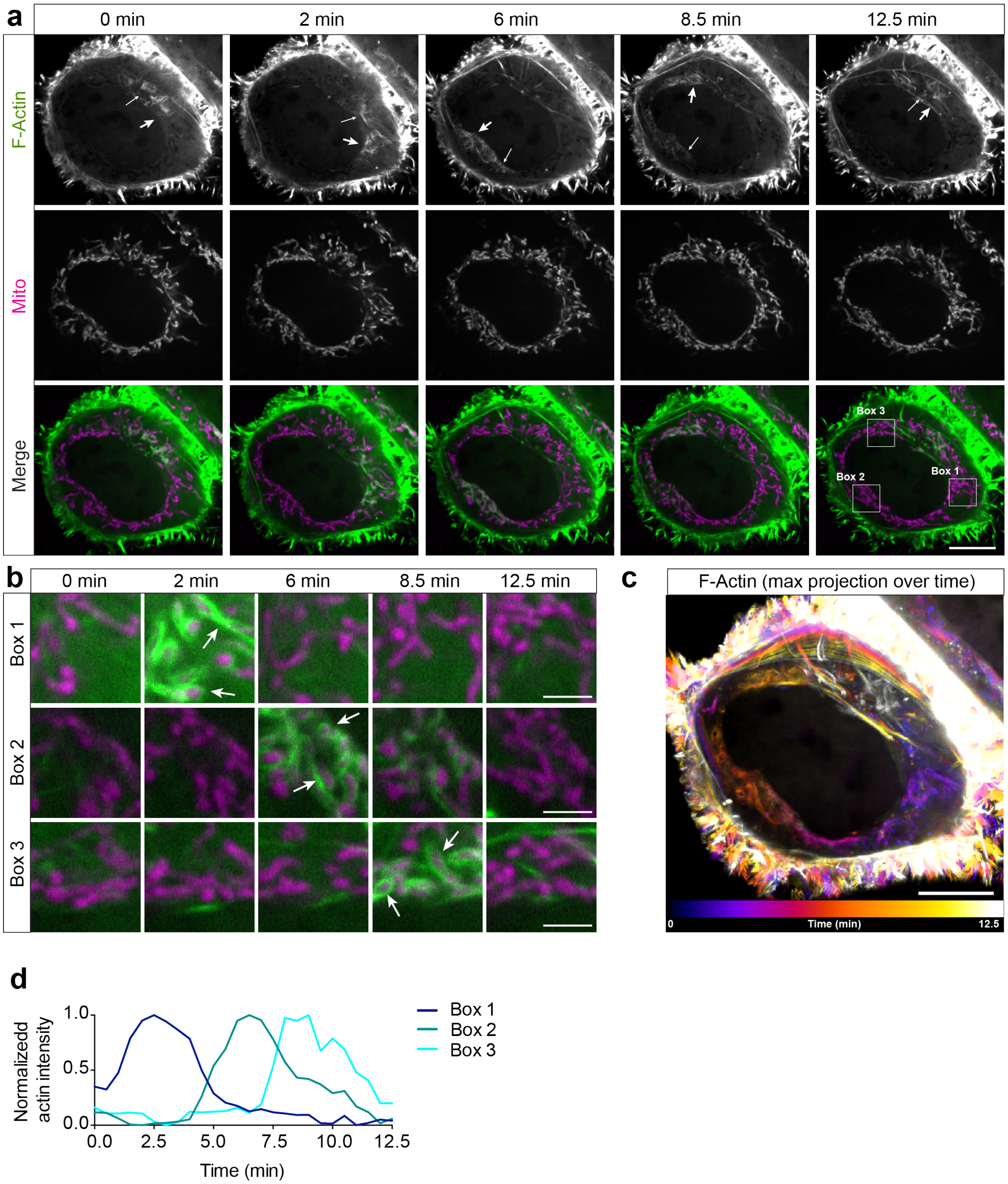


**Supplementary Figure 3. F-actin cycles through mitochondrial subpopulations.**

**(a)** Confocal time series of F-actin (LifeAct-GFP) cycling through subpopulations of mitochondria (Mito-DsRed2). **(b)** Enlarged images of Boxes 1-3. White arrows indicate actin-positive mitochondria. **(c)** Pseudocolored maximum intensity projection of F-actin localization over 12.5 min. **(d)** Normalized intensity of LifeAct-GFP in boxes 1-3 over time. Scale bars (**a**,**c**), 10 μm; (**b**), 2.5 μm

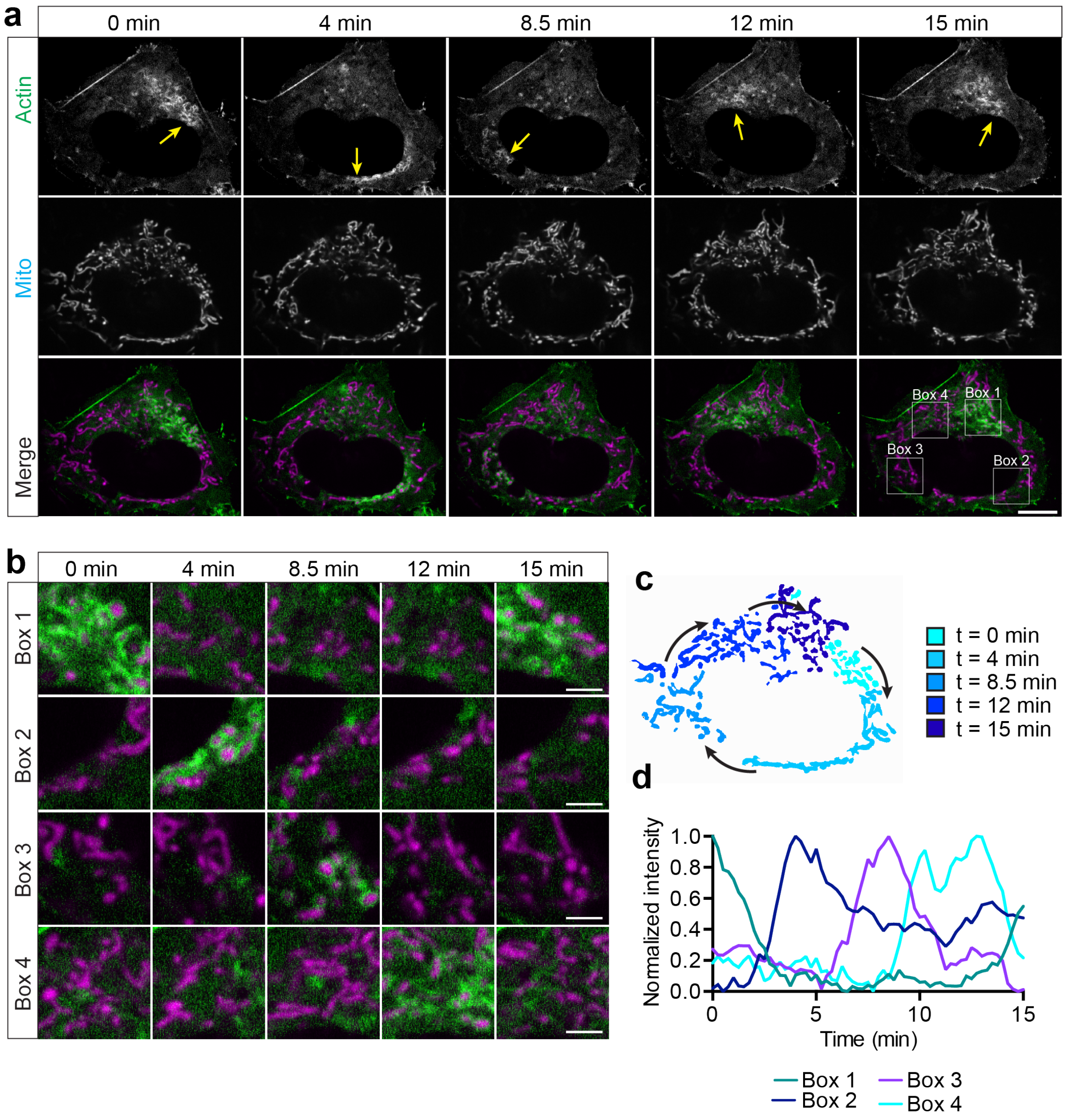


**Supplementary Figure 4. Actin cycles through mitochondrial subpopulation over time.**

(**a**) Confocal time series of actin (GFP-actin) recruitment to distinct populations of mitochondria (Mito-DsRed2) over 15 min. **(b)** Enlarged images of boxes 1-4 displaying ordered actin assembly onto and disassembly off of individual mitochondria. **(c)** Model depicting actin cycling in a persistent clockwise direction, sampling all mitochondria within the cell. **(d)** Normalized intensity of GFP-actin in boxes 1-4 over 15 min. Scale bars (**a**), 10 µm; (**b**), 2.5 µm.

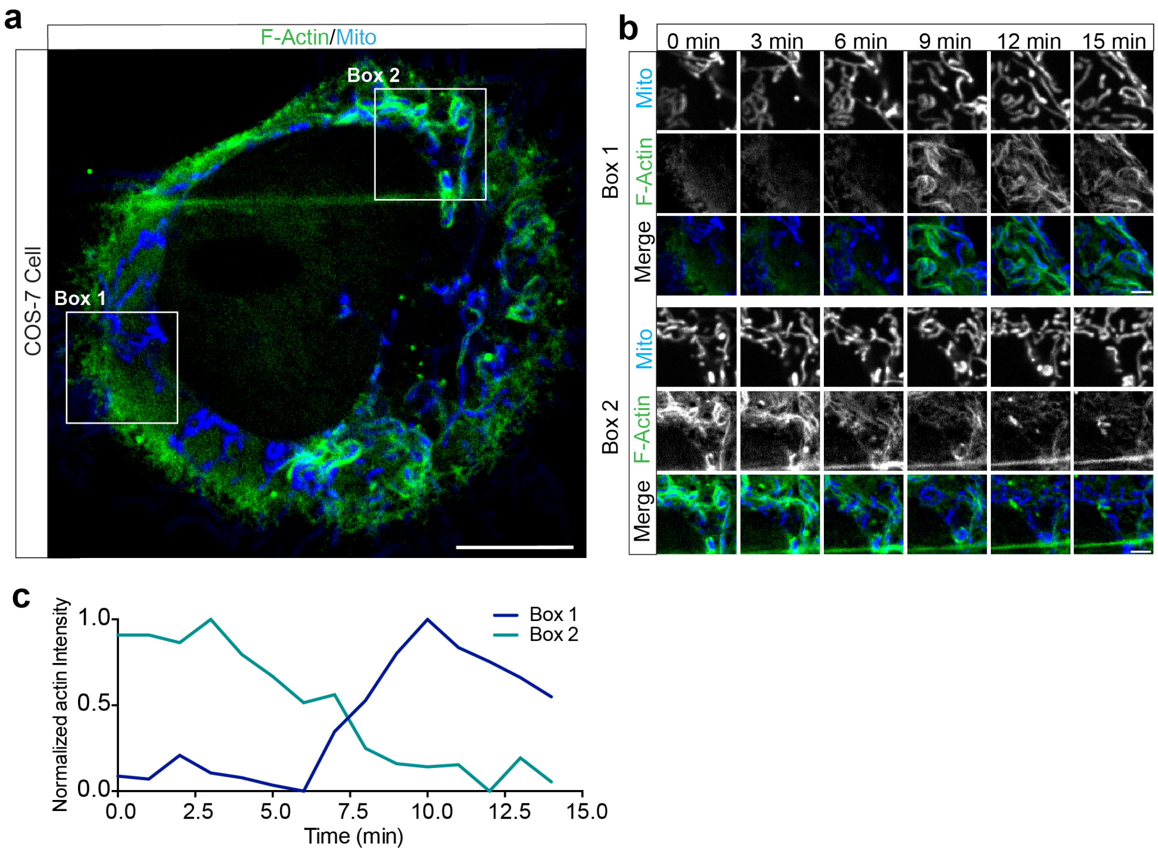


**Supplementary Figure 5. Actin cycles through mitochondrial subpopulations in Cos-7 cells.**

**(a)** Confocal image of F-actin (LifeAct-GFP) recruitment to mitochondria (Mito-DsRed2) in Cos-7 cells. **(b)** Enlarged images of Boxes 1-2, indicating actin polymerization onto mitochondria in Box 1 and depolymerization from mitochondria in Box 2 over 15 min. **(c)** Normalized intensity of LifeAct-GFP in Boxes 1 and 2 over 14 min. Scale bars (**a**), 10 µm; (**b**), 2.5 µm.

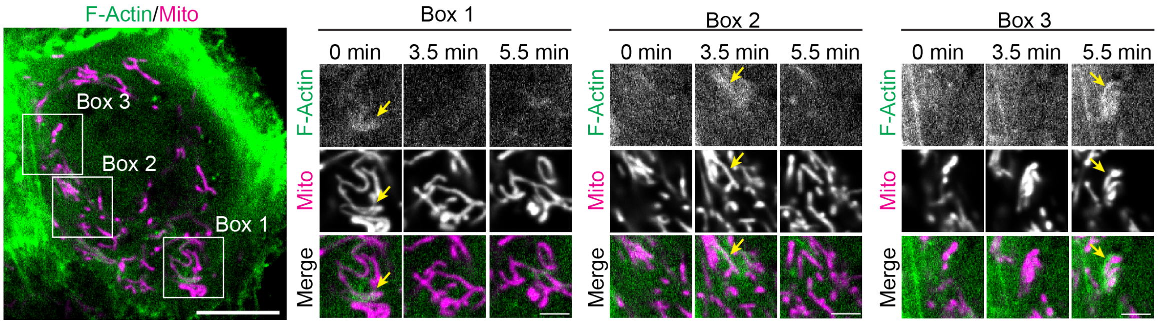


**Supplementary Figure 6. Actin cycles through mitochondrial subpopulations in normal human epidermal keratinocytes**

Confocal image of normal human epidermal keratinocyte (NHEK) expressing LifeAct-GFP and Mito-DsRed2 (left). F-actin cycles from mitochondria in Box 1 to Box 2 over 3.5 min. Between 3.5 and 5.5 min, actin cycles from mitochondria in Box 2 to Box 3. Yellow arrows indicate actin-positive mitochondria. Scale bars (left) 10 µm; (right), 2.5 µm.

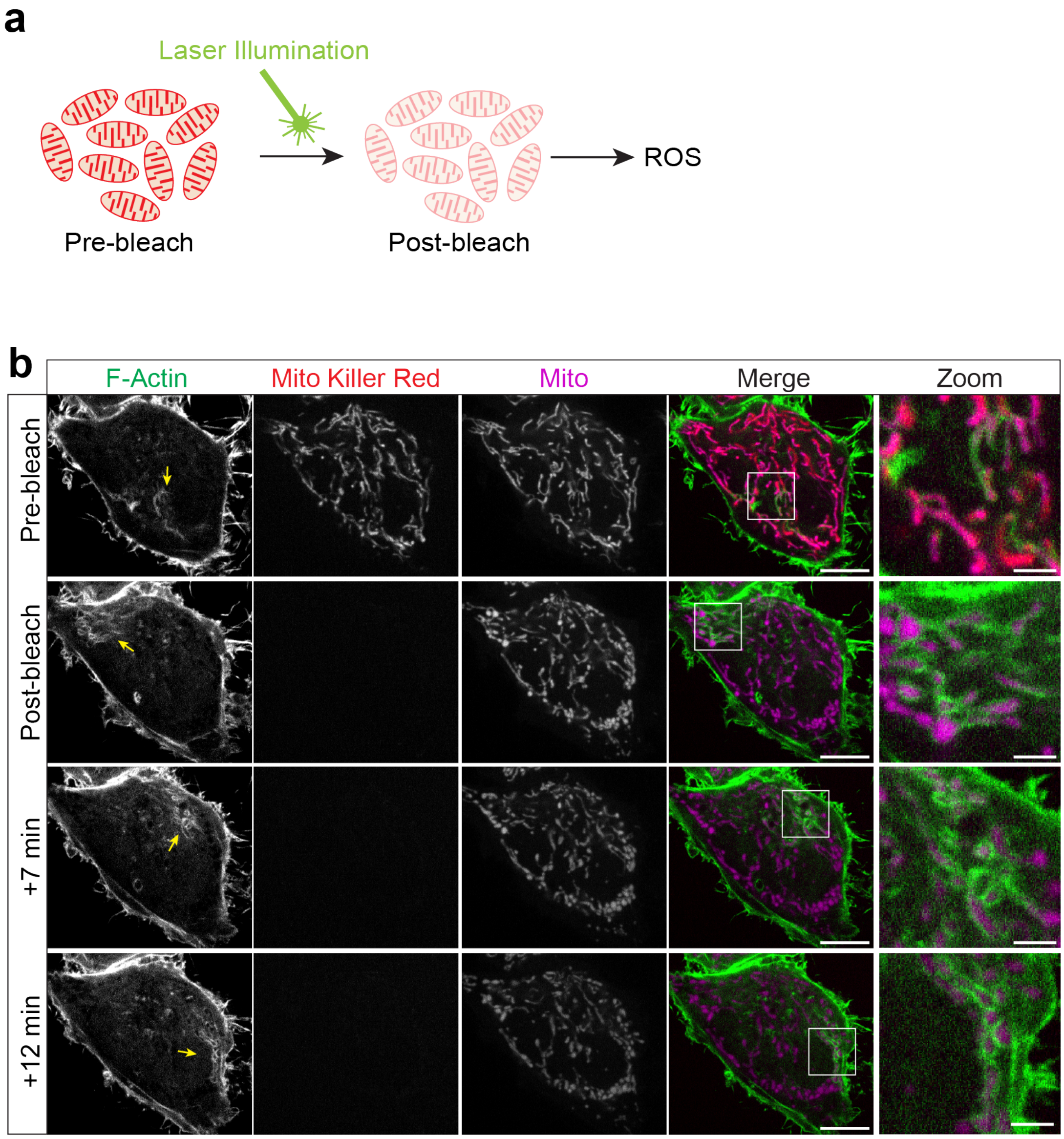


**Supplementary Figure 7. Increased ROS production does not disrupt actin cycling through mitochondrial subpopulations.**

(**a**) Model: Illumination of mitochondrial-targeted Mito Killer Red by 561 nm laser light generates reactive oxygen species (ROS) within the mitochondrial matrix. (**b**) Confocal time series of HeLa cell expressing LifeAct-GFP, Mito-BFP, and Mito Killer Red. Activation of Mito Killer Red by photobleaching does not impede actin cycling over time. Yellow arrow indicates subpopulation of actin-positive mitochondria. Scale bars (**b**, full size), 10 μm, (**b**, zoom), 2.5 μm.

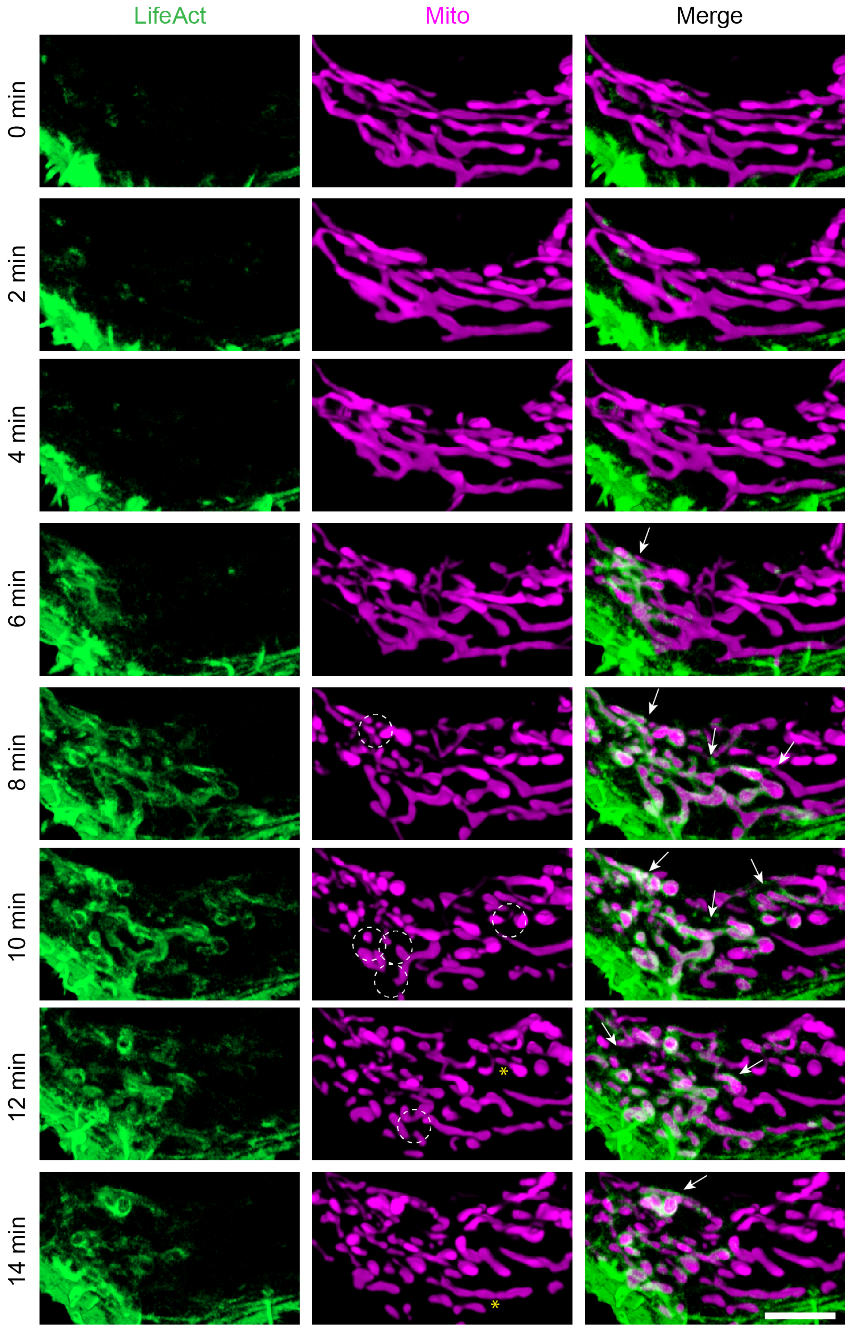


**Supplementary Figure 8. Actin polymerization induces mitochondrial fission and local mitochondrial network remodeling.**

Time lapse 3D-renderings of HeLa cell mitochondria over 14 min. Mito-DsRed2 labeled mitochondria within the displayed subregion are initially elongated and interconnected. As F-actin (LifeAct-GFP) cycles onto the elongated mitochondria (white arrows) the mitochondria undergo robust fission. Dashed circles indicate sites of mitochondrial fission. By 14 min, actin has cycled off of the mitochondria in the right-hand side of the window, and the fragmented mitochondria begin to fuse (yellow asterisks) and recover their tubular morphology. See Fig. 6c for enlarged max projection of right hand region. Scale bar 5 μm.

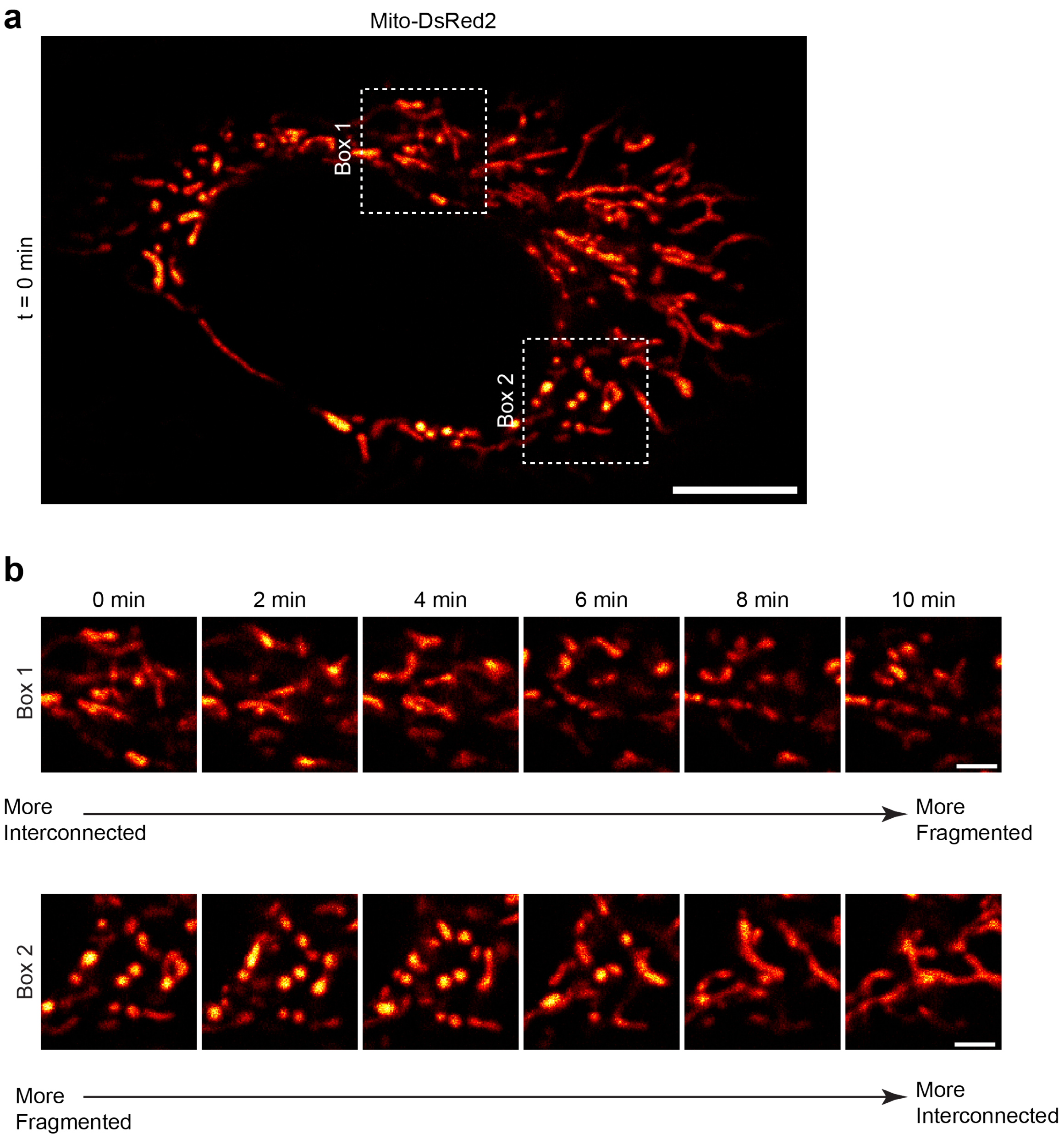


**Supplementary Figure 9. Mitochondrial networks undergo simultaneous growth and fragmentation in a single cell.**

(**a**) Confocal image of HeLa cell transfected with Mito-DsRed2. Box 1 indicates a subregion of the mitochondrial network that is more interconnected, while Box 2 indicates a subregion of the mitochondrial network that is more fragmented. (**b**) Mitochondria within Box 1 become increasingly fragmented over ten min, while mitochondria in Box 2 become increasingly interconnected. Scale bar (**a**) 10 μm; (**b**) 2.5 μm.

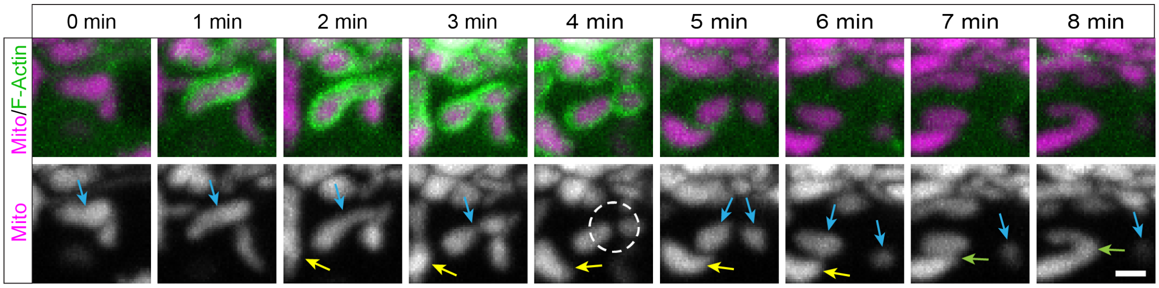


**Supplementary Figure 10. Fragmented mitochondria rapidly fuse after actin depolymerization.**

Confocal time series indicating F-actin (LifeAct-GFP) cycling onto and off of an individual Mito-DsRed2 labeled mitochondrion (blue arrow). Over 4 min, actin polymerization promotes fission of the indicated mitochondrion (dashed circle, t=4 min), resulting in two daughter mitochondria. Over the subsequent 4 min, one daughter mitochondrion (left blue arrow) comes into close contact with a separate, adjacent mitochondrion (yellow arrow), eventually fusing (green arrow). Over the same time period, the other daughter mitochondrion remains fragmented (right blue arrow). Scale bar 1 µm.

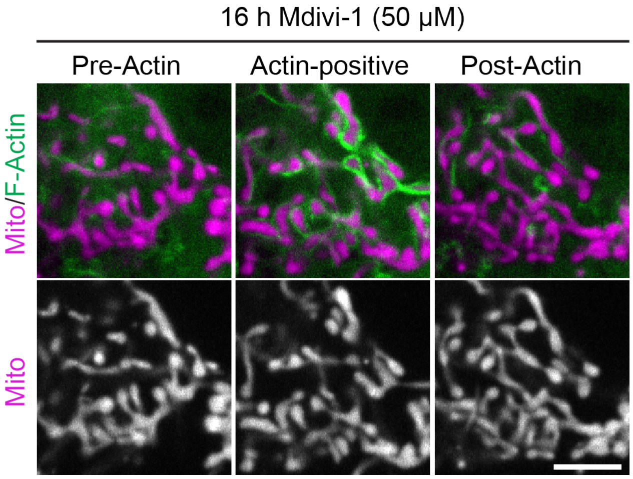


**Supplementary Figure 11. Actin does not promote mitochondrial fragmentation upon Drp1 inhibition.**

Maximum intensity projection of mitochondria subpopulation in HeLa cell treated with the Drp1 inhibitor Mdivi-1 (50 µM for 16 h). F-actin (LifeAct-GFP) assembles on elongated mitochondria (Mito-DsRed2), but does not promote robust fragmentation. Scale bar, 5 µm.
